# Niosomal Encapsulation of *Oroxylum indicum* Leaf Extract for Topical Anti-Inflammatory Application

**DOI:** 10.3390/pharmaceutics18080955

**Published:** 2026-08-03

**Authors:** Pattaraphorn Panomai, Nattawadee Kanpipit, Natsajee Nualkaew, Suthasinee Thapphasaraphong

**Affiliations:** 1 Health Science and Aesthetic Program, Faculty of Science and Technology, Rajamangala University of Technology Krungthep, Bangkok 10120, Thailand; pattaraphorn.p@mail.rmutk.ac.th; 2 Faculty of Pharmaceutical Sciences, Khon Kaen University, Khon Kaen 40002, Thailand; natawadee.k@kkumail.com; 3 Department of Pharmacognosy and Toxicology, Faculty of Pharmaceutical Sciences, Khon Kaen University, Khon Kaen 40002, Thailand; nnatsa@kku.ac.th; 4 Department of Pharmaceutical Chemistry, Faculty of Pharmaceutical Sciences, Khon Kaen University, Khon Kaen 40002, Thailand

**Keywords:** *Oroxylum indicum*, flavonoids, nanovesicles, in vitro permeation, controlled release, nitric oxide inhibition, topical anti-inflammatory

## Abstract

**Background:** *Oroxylum indicum* (L.) Kurz is a medicinal plant widely used in traditional Thai medicine, reported to exhibit anti-inflammatory activity. However, its topical use is limited by the poor dermal delivery of its active compounds. This study aimed to develop and characterize a topical niosomal delivery system containing *O. indicum* leaf extract to enhance permeation through Strat-M^®^ membrane and anti-inflammatory activity. **Methods:** Extract-loaded niosomes were prepared via thin-film hydration using non-ionic surfactants and cholesterol. The developed niosomes were evaluated for their physicochemical properties, in vitro release and in vitro permeation, stability, and anti-inflammatory effects in LPS-stimulated RAW 264.7 cells. **Results:** The optimal formulation consisted of a phosphate buffer at pH 5.5, Span 60, cholesterol, 0.5% (*w*/*v*) extract, and 10% propylene glycol, with the extract added during the hydration step. The optimal formulation showed a high encapsulation efficiency (>70% (total phenolics) and >90% (total flavonoids), a nano-sized particle size of approximately 100–200 nm with a narrow size distribution, and a zeta potential within the acceptable value (≤−30 mV). The successful incorporation of the extract into niosome bilayers was confirmed by FTIR spectroscopy. The niosomal formulation demonstrated a significantly more sustained and controlled release of total phenolics and flavonoids, including enhanced permeation of phenolic compounds across the Strat-M^®^ membrane, compared to the extract solution. Formulations containing 0.3–0.5% extract remained physically stable, maintaining encapsulation efficiency, particle size, and zeta potential under thermal stress conditions. Significantly, niosomes loaded with 0.5% extract exhibited the greatest inhibition of nitric oxide production in RAW 264.7 cells without cytotoxicity. **Conclusions:** These findings are based on in vitro membrane permeation and cell-based assays; further ex vivo or in vivo skin studies are required to confirm topical anti-inflammatory efficacy.

## 1. Introduction

*Oroxylum indicum* (L.) Kurz is a medicinal plant widely used in traditional Thai medicine, reported to possess anti-inflammatory, anti-diarrheal, anti-arthritic, and wound treatments [1]. The major bioactive constituents identified in various parts of *O. indicum* include the flavonoids baicalin, baicalein, chrysin, and oroxylin A, which have been reported to exhibit anti-inflammatory, antioxidant, antimicrobial, and anticancer activities [2]. However, some of these phytochemicals possess limited aqueous solubility and stability, posing challenges for their topical delivery and permeation across biological barriers [3]. The primary bioactive compounds found in various parts of *O. indicum* are flavonoids, including non-polar substances such as oroxylin A and chrysin, which present challenges in terms of release and skin penetration in topical formulations. The leaves, stalks, and pods of *O. indicum*, which contain similar phytochemical components, have been identified as alternative raw materials for exploring their potential anti-inflammatory effects [4]. Our previous study highlighted that the leaf extract exhibits superior bioactive potential, particularly stronger anti-inflammatory activity, and analyzed its major active flavonoids. In addition, *O. indicum* leaf extract exhibits significant anti-inflammatory activity, primarily by inhibiting nitric oxide (NO) production in macrophages [5]. However, the application of phytochemicals is restricted because of their susceptibility to environmental factors, including temperature, pH, and light intensity, which can accelerate their degradation and create challenges in preserving their potency [6].

Furthermore, their ability to permeate the skin is limited. For a substance to permeate the skin, it must first pass through the epidermis, which is a layer of dead skin cells that acts as a barrier. Substances capable of penetrating the skin must possess the ability to dissolve or pass through the gaps between skin cells or intercellular spaces [7]. Therefore, niosomes were selected as the topical delivery system because they can improve the permeation of active compounds while enhancing their physicochemical stability during storage [8,9]. Several topical niosomal formulations containing herbal extracts with anti-inflammatory activity have been reported to demonstrate improved skin permeation, chemical stability, and topical efficacy compared with conventional formulations. For instance, niosomal gels of *Zingiber cassumunar* extract and ginger-based preparations have demonstrated enhanced dermal delivery and sustained anti-inflammatory effects [10]. However, no niosomal system has been developed for *O. indicum*, and previous studies have rarely provided an integrated evaluation of physicochemical characterization, in vitro permeation, and in vitro anti-inflammatory activity within a single formulation.

This study aimed to develop and evaluate, for the first time, a niosome-based topical formulation encapsulating *O. indicum* leaf extract using a comprehensive approach that integrates formulation optimization, physicochemical characterization, release kinetics, in vitro permeation, stability assessment, and in vitro anti-inflammatory activity. Niosomes were prepared using the thin-film hydration method and optimized by varying the surfactant type (Span 20, 60, and 80), pH, co-solvent type, and extract concentration. The resulting formulations were characterized in terms of particle size, polydispersity index (PDI), encapsulation efficiency, and morphology, and were further evaluated for drug release, in vitro permeation, and in vitro anti-inflammatory activity.

## 2. Materials and Methods

### 2.1. Materials

The following chemicals and reagents were used in this study Methanol (RCL Labscan, Bangkok, Thailand), acetonitrile HPLC grade (RCL Labscan, Bangkok, Thailand), quercetin, and gallic acid (St. Louis, MO, USA), baicalin, baicalein, chrysin, and oroxylin A were purchased from Biopurify (Chengdu, China). Other reagents used included sodium carbonate (Fisher Scientific, Loughborough, UK). Folin–Ciocalteau reagent (Srichem, Mumbai, India), N (ω)-nitro-L-arginine methyl ester (L-NAME), sodium nitrite (Sigma-Aldrich, St. Louis, MO, USA), lipopolysaccharide (LPS; Sigma-Aldrich, Darmstadt, Germany), N-(1-naphthyl) ethylenediamine dihydrochloride (NED; PanReac, Darmstadt, Germany), Dulbecco’s modified Eagle’s medium (DMEM) (Hyclone, Logan, UT, USA), penicillin-streptomycin (Hyclone, Logan, UT, USA), fetal bovine serum, (FBS) (Hyclone, Logan, UT, USA), dimethyl sulfoxide (DMSO) (Amresco, Solon, OH, USA), 0.25% trypsin-EDTA (GibCo, Mississauga, Canada), trypan blue (Gibco, Grand Island, NY, USA), 3-(4,5-dimethylthiazol-2-yl)-2,5-diphenyltetrazolium bromide (MTT) (Gibco, Grand Island, NY, USA). Non-ionic surfactants, such as Span 20 (SP20), Span 60 (SP60), and Span 80 (SP80), along with cholesterol (CH), phosphate-buffered saline (PBS 5.5, 6.4, 7.4), polyethylene glycol 400 (PEG400), propylene glycol (PG), and dialysis membrane (molecular weight cut-off 14,000 kDa) were also used. All reagents and chemicals used in this study were of analytical grade.

### 2.2. Method

#### 2.2.1. Preparation of Plant Materials and Sample Extraction

The *O. indicum* plant leaves were collected from Bueng Kan Province, Thailand, between May and July 2020. The botanical identity was officially authenticated, and the specimens were deposited at the Herbarium of the Faculty of Pharmaceutical Sciences, Khon Kaen University, Thailand (Voucher Specimen No. 3695-3690). The leaves chosen for extraction underwent specific selection. They were cleaned and dried in a hot-air oven at 50 °C for 72 h for three days, then ground into a fine powder. A total of 150 g of this powder was macerated with 80% ethanol in a 1:10 *w*/*v* ratio for seven days. The mixture was then filtered and evaporated to eliminate the solvent. The extract was pre-freeze-dried at –80 °C for 72 h using a lyophilizer to obtain dry powder. The extract was characterized by determining its total phenolic and flavonoid contents. In addition, the chemical markers baicalin, baicalein, chrysin, and oroxylin A were quantified using high-performance liquid chromatography (HPLC) according to the validated method described in our previous study. Briefly, the analysis was performed on a C18 reversed-phase column under gradient elution with water (0.1% formic acid in water) and acetonitrile (0.1% formic acid in acetonitrile) with UV detection at 254 nm. Quantification was performed using external calibration curves for each reference standard [5].

#### 2.2.2. Preparation of *O. indicum* Leaf Extract in Niosome

The niosomal formulations were prepared using the thin-film hydration method, maintaining a fixed surfactant-to-cholesterol molar ratio of 1:1 (mM). The compositions of the 12 formulations, which varied in phosphate-buffered solution (PBS) pH (5.5, 6.5, or 7.4), surfactant type (Span 20, Span 60, or Span 80), and the presence of 5 or 10% PEG 400 or propylene glycol (PG), are listed in Table 1.

Briefly, the required amounts of 0.1% (*w*/*v*) crude extract, non-ionic surfactants, and cholesterol for each formulation were dissolved in 10 mL of 95% ethanol and heated in a water bath at 55–60 °C for approximately 1 min. The solvent was then evaporated using a rotary evaporator at 30 rpm, 150–60 mbar, and 45 °C, allowing a uniform thin film to form on the inner wall of the flask.

The thin film was subsequently hydrated with 10 mL of aqueous medium (deionized water or PBS buffer at pH 5.5, 6.5, or 7.4) containing PEG 400 or PG, as specified for F1–11 in Table 1. In F12, the plant extract at a concentration of 0.1% (*w*/*v*) was incorporated during the hydration step by dissolving it in 10% PG in PBS (pH 5.5). Hydration was performed using a rotary evaporator at 60 °C and 100 rpm for 30 min, and the detachment of the thin film was visually confirmed.

The niosomal dispersion was sonicated in a bath sonicator for 30 min to reduce vesicle size and improve homogeneity. All formulations were further characterized based on their physicochemical properties. The criteria for choosing the optimal formulation were an encapsulation efficiency exceeding 70%, mean particle size below 500 nm, a polydispersity index (PDI) below 0.5, and a zeta potential of ≥+30 mV or <−30 mV to ensure adequate physical stability [11].

After the optimal formulation had been selected using a fixed extract concentration of 0.1% (*w*/*v*), the effect of extract loading was further investigated. Six formulations were prepared following the same procedure, varying only the extract concentration from 0 to 0.5% (*w*/*v*).

#### 2.2.3. Physicochemical Characterization

The encapsulation efficiency of the niosomes was evaluated by separating the pellet from the supernatant through centrifugation using a Vivaspin^®^ centrifugal concentrator (Sartorius AG, Göttingen, Germany). The niosomes were centrifuged at 5000× *g* and 4 °C for 30 min. After discarding the supernatant, a methanol solution was added to the niosome residues. The dissolved solution was then analyzed to determine the total phenolic content (TPC) and flavonoid content (TFC) using gallic acid and quercetin as reference standards. The encapsulation efficiency (%EE) was calculated from the TPC or TFC values using Equation (1).

(1) $$\mathrm{Encapsulation}\;\mathrm{Efficiency}\;(\%\mathrm{EE})=\frac{Amount\;of\;Phenolic\;or\;Flavonoid\;Compounds\;from\;pellet.}{Total\;amount\;of\;Phenolic\;or\;Flavonoid\;Compounds}$$

The physicochemical characteristics of the niosomes were analyzed using dynamic light scattering (DLS). The particle size, polydispersity index (PDI), and zeta potential of the formulations were evaluated. Particle size analysis was conducted using a Zetasizer (Zeta Sizer Nano ZS, Malvern Instruments Ltd., Malvern, UK). For zeta potential measurement, the sample was diluted at a 1:100 ratio with water, whereas nanoparticle size and dispersion measurements required dilution at a 1:100 ratio with PBS. The prepared samples were placed in 1.5 mL microtubes, and 700 µL of each sample was transferred to a cuvette for further analyses. The cuvette was inserted into the Zetasizer, which provided the zeta potential in millivolts (mV), particle size in nanometers (nm), and polydispersity index of niosomes [12].

#### 2.2.4. Stability Evaluation Under Temperature Cycling Conditions

The stability of the niosomes was evaluated by incubating the samples at 4 °C in a refrigerator and 45 °C in a hot air oven. Each cycle consisted of 24 h incubation at each temperature, with two temperature switches per cycle, repeated for six cycles. The samples were then analyzed for percentage encapsulation efficiency, polydispersity index (PDI), and zeta potential. Stability was determined by comparing the total combined content after the cycles to the initial content measured on day 0 [13].

#### 2.2.5. Transmission Electron Microscopy (TEM)

A 10 µL volume was deposited on each sample using a carbon-coated copper grid. The samples were air-dried for 2–4 h before being analyzed using TEM (Tecnai G2 20, FEI Company, Hillsboro, OR, USA) at the central laboratory of the Faculty of Science, Khon Kaen University. The results are displayed as images [14].

#### 2.2.6. Attenuated Total Reflectance (ATR)-Fourier Transform Spectroscopy (FTIR) Analysis

The formulations were analyzed using Fourier-transform infrared (FTIR) spectrophotometry (Bruker Tensor II Series; Billerica, MA, USA). Blank niosomes and sample niosomes were placed on the crystal surface of the spectrophotometer. Subsequently, cold air was used for 10 min to dry the sample. The analysis was performed over the spectral range of 4000–400 cm^−1^ with a 4 cm^−1^ resolution for 64 co-added scans per spectrum [12].

#### 2.2.7. In Vitro Release Study and Kinetic Modeling

The release profiles of the niosomes were evaluated based on the phenolic and flavonoid contents at various time intervals. The samples were prepared using a Franz diffusion cell setup with a dialysis membrane (cut-off 14,000 Da) in the receptor compartment of the cell. This compartment was filled with 5 mL of 40% ethanol in PBS at pH 7.4 (*v*/*v*), and a volume of 5 mL was used. The donor compartment containing suspended niosome extracts of the target substances was placed on the donor side, and 1 mL of the volume was added. The Franz diffusion cell system was maintained at a controlled temperature of 37 °C and stirring speed of 800 rpm throughout the experiment. Samples (0.6 mL) were collected from the receptor at 0.5, 1, 2, 4, 6, 8, 12, and 24 h, and the receptor was replaced with 40% ethanol in PBS at pH 7.4. The collected samples were subsequently analyzed for their phenolic and flavonoid contents. The release profiles of the four major flavonoid markers (baicalin, baicalein, chrysin, and oroxylin A) were also determined by HPLC. The data were reported as µg/mL and calculated as the cumulative release of total phenolic and flavonoid contents, including these four chemical markers [15]. The data on the release were studied to investigate the release mechanisms and compare the different release mechanisms. These mechanisms include zero-order kinetics, first-order kinetics, Higuchi’s model, and Korsmeyer–Peppas model. The models were calculated using the following equations [16].

#### 2.2.8. In Vitro Permeation Study

The permeation of niosomes was evaluated by measuring the phenolic and flavonoid contents that permeated across a Strat-M membrane into the receptor compartment of Franz diffusion cell at predetermined time intervals. The receptor compartment was filled with 40% ethanol in PBS at pH 7.4 (*v*/*v*) with a volume of 5 mL. The donor compartment containing suspended niosome extracts of the target substances was placed on the donor, and the volume was set to 1 mL. The Franz diffusion cell system was maintained at a controlled temperature of 37 °C, and a spin speed of 800 rpm throughout the experiment, and 0.5 mL of samples were collected from the receptor at 0.5, 1, 2, 4, 6, 8, 12, and 24 h. It was then replaced with 40% ethanol in PBS at pH 7.4 (*v*/*v*). The collected samples were subsequently analyzed for their phenolic content. The cumulative percentage and time were constructed, and the cumulative permeation steady-state flux (JSS) was determined from the slope of the linear regression. The lag time (T/lag) was obtained using the linear area of the *x*-axis intercept of the cumulative permeation profile. The permeability coefficient (P) and enhancement ratio (ER) were calculated using Equations (2) and (3), respectively [17].

P = J_SS_/C_0_ (Initial concentration) (2)

ER = J_SS_ test/J_SS_ control (3)

### 2.3. Evaluation of Anti-Inflammatory Activity in RAW 264.7 Cells

#### 2.3.1. Cell Viability

The murine leukemia macrophage cell line (RAW 264.7) was maintained in Dulbecco’s modified Eagle’s medium (DMEM) mixed with 10% FBS and 1% penicillin-streptomycin and incubated at 37 °C in a 5% CO_2_ atmosphere.

RAW 264.7 cells (ATCC TIB-71™, murine macrophages, Mus musculus) were seeded in 96-well plates at a density of 1 × 10^4^ cells/well and incubated at 37 °C in a 5% CO_2_ atmosphere for 24 h. Cell viability was measured by mitochondrial reduction of MTT. *O. indicum* leaf extract solution and niosomal formulations were dissolved in DMEM and used to treat cells. Untreated cells cultured in DMEM with 10% FBS served as the (-) control. After 24 h of incubation, the medium was replaced with 0.5 mg/mL MTT solution in PBS (pH 7.4) and incubated for 4 h. The solution was then removed, and the formazan crystals were dissolved in 100 µL/well of DMSO. Absorbance was measured at 570 nm using a microplate reader. Cell viability was calculated using Equation (4).

% Cell viability = (As/Ac) × 100(4)

where Ac is the absorbance of the (-) control and As is the absorbance of the treated cells.

#### 2.3.2. Nitric Oxide Inhibition

RAW 264.7 cells were seeded in 96-well plates at a density of 1 × 10^4^ cells/well and incubated at 37 °C in a 5% CO_2_ atmosphere for 24 h. The medium in each well was replaced with DMEM containing various concentrations of *O. indicum* leaf extracts, niosome formulations, and 100 ng/mL of LPS for 24 h. Treatment with 250 µM N-nitro-L-arginine methyl ester (L-NAME) was used as a positive control, and LPS-treated cells served as the control. After treatment, the media were mixed with 100 µL of Griess reagent in each well and incubated for 10 min at room temperature. Absorbance was measured at 540 nm using a microplate reader. Nitric oxide inhibition was calculated as a percentage using Equation (5).

% NO inhibition = [(A_control_ − A_sample_)/A_control_] × 100 (5)

A_control_ = Absorbance from the untreated cells

A_Sample_ = Absorbance from sample-treated cells (6)

### 2.4. Statistical Analysis

All experiments were conducted, and the results are expressed as mean ± SD. Statistical analyses were performed using one-way analysis of variance (ANOVA) in SPSS version 29.0 (IBM Corp., Armonk, NY, USA) with Tukey’s post hoc test or Student’s *t*-test as appropriate. A *p*-value of less than 0.05 was considered statistically significant. Error bars and significance levels are detailed in the figure legends.

## 3. Results

### 3.1. The Physicochemical Characterization

#### 3.1.1. Phytochemical Characterization of the *O. indicum* Extract

The crude *O. indicum* extract contained 15.96 ± 4.45 mg GAE/g DW of total phenolic content (TPC) and 6.31 ± 1.96 mg QE/g DW of total flavonoid content (TFC) (Table S3), confirming the presence of phenolic and flavonoid constituents before niosome formulation. The results of phytochemical markers in the *O. indicum* extract were also characterized using a validated HPLC method summarized in Table S1. The HPLC chromatogram of mixed standards of baicalein. Baicalein, Chrysin and Oroxylin A was in Figure S1, and the standard curves of 4 markers were in Figure S2. Quantitative analysis showed that baicalin (33.44 ± 4.91 µg/mg extract) was the predominant flavonoid marker, followed by baicalein (27.50 ± 8.23 µg/mg extract), chrysin (5.20 ± 2.44 µg/mg extract), and oroxylin A (24.06 ± 7.20 µg/mg extract) (Table S2).

#### 3.1.2. Characterization of Niosomes Loaded with *O. indicum* Leaf Extracts

This study systematically evaluated the effects of pH, non-ionic surfactants, and co-solvents on the encapsulation efficiency and physicochemical characteristics of niosomes. Twelve formulations containing *O. indicum* leaf extract were prepared (Table 1), and their physicochemical characteristics are shown in Table 2.

When comparing the encapsulation efficiency, particle size, and zeta potential across different phosphate buffer pH conditions (Table 2), F2 (pH 5.5) exhibited the highest encapsulation efficiency, with 31.38 ± 2.10% for TPC and 57.34 ± 6.40% for TFC (Table 2). In terms of particle size, F2 (pH 5.5) exhibited the smallest particle size (201.33 ± 37.79 nm), followed by F4 (pH 7.4; 232.67 ± 53.46 nm), F1 (DI water; 262.63 ± 57.39 nm), and F3 (pH 6.5; 282.20 ± 8.29 nm). Regarding size distribution, F4 demonstrated the lowest PDI value (0.43 ± 0.04), indicating the most uniform size distribution, followed by F2 (0.49 ± 0.01), F3 (0.69 ± 0.03), and F1 (1.00 ± 0.00). All formulations exhibited zeta potential values below −30 mV, except for F1 (0.97 ± 3.56 mV). Specifically, F2, 3, and 4 exhibited zeta potential values of −41.93 ± 15.39 mV, −37.27 ± 1.40 mV, and −44.60 ± 6.29 mV, respectively, indicating good stability.

The results obtained using various surfactants indicated that F5 (Span 20) and F6 (Span 60) achieved high encapsulation efficiencies for TPC, at 49.99 ± 1.45% and 46.46 ± 4.27%, respectively, which were significantly higher than that of F7 (Span 80), which exhibited an encapsulation efficiency of 14.24 ± 10.85% (Table 2).

In terms of particle size, F6 (Span 60) demonstrated a smaller particle size (292.33 ± 41.20 nm) than F5 (590.67 ± 18.93 nm) and F7 (487.33 ± 45.17 nm). Additionally, F6 showed a lower PDI (0.63 ± 0.10), indicating a more uniform size distribution than that of F5 and F7, both of which had a PDI of (1.00 ± 0.00).

Regarding Zeta potential values, all formulations containing Span 20, 60, and 80 exhibited values below −30 mV, specifically −58.46 ± 5.06, −48.53 ± 4.21, and −35 ± 0.85 mV, respectively, indicating good stability. Although non-ionic surfactants were used, the negative zeta potential arose from the ionization of acidic functional groups in the extract and buffer, revealing a net negative surface charge on the niosomes.

Additive co-solvents, such as polyethylene glycol 400 (PEG400) and propylene glycol (PG), were incorporated to minimize niosome coalescence. The results indicated that formulations containing PG exhibited higher encapsulation efficiencies for total phenolic content (TPC) and total flavonoid content (TFC) than those containing PEG400.

F8 and F9, containing 5% and 10% PEG400, respectively, achieved TPC encapsulation efficiencies of 41.98 ± 2.06% and 11.78 ± 2.94%, and TFC encapsulation efficiencies of 67.21 ± 0.96% and 24.31 ± 2.87%, respectively. In contrast, F10 and F11, containing 5% and 10% PG, respectively, demonstrated higher encapsulation efficiencies for TPC at 64.14 ± 1.40% and 70.55 ± 1.02%, respectively, and for TFC at 81.51 ± 11.82% and 86.12 ± 8.51%, respectively (Table 2). However, F12 achieved the highest encapsulation efficiencies for TPC and TFC, at 67.98 ± 1.17% and 89.52 ± 3.53%, respectively, when the extract was added during hydration. This suggests that the timing of extraction significantly influences the encapsulation efficiency [18].

The addition of PEG 400 or PG to niosome formulations significantly influenced the particle size. The particle size of formulations F8–F11 ranged from 277.80 to 800.53 nm, with PDI values between 0.24 and 1.00 (Table 2). Formulations containing PG at equivalent concentrations exhibited larger vesicle sizes than those containing PEG400. Increasing the concentration of co-solvents from 5% to 10% significantly reduced the particle size. Specifically, niosomes with 10% PG (F11) displayed a smaller particle size (277.80 ± 16.66 nm) than those with 5% PG (F10), which measured 800.53 ± 14.48 nm. A similar trend was observed for PEG400; niosomes containing 10% PEG400 (F8) exhibited smaller vesicles at 239.00 ± 2.48 nm compared to those containing 5% PEG400 (F9), which showed a vesicle size of 504.67 ± 33.50 nm. Notably, when the extract was incorporated during the hydration step in F12, the smallest particle size of 174.50 ± 13.16 nm was obtained (Table 2).

The zeta potential values of the most niosome formulations (F2–F12) were below −30 mV, indicating good stability. The only exception was F9 (−27.87 ± 1.62 mV). However, for F12, the zeta potential was also acceptable (−34.53 ± 1.15 mV), as shown in Table 2.

The selected formulation from Table 2 was F12, which demonstrated the most suitable composition, consisting of a pH of 5.5, Span 60, and 10% PG. Further experiments were conducted to determine the optimal concentration of *O. indicum* leaf extract required in the formulation to achieve the desired niosome characteristics.

Based on the study of suitable concentrations of *O. indicum* leaf extract, most niosome formulations containing 0.1–0.5% extract achieved TPC encapsulation efficiencies of 52.83–70.88%, while TFC encapsulation efficiencies were above 74% (Table 3). All formulations demonstrated higher encapsulation efficiencies for TFC than for TPC in this study. Consequently, increasing the concentration of *O. indicum* leaf extract tended to enhance the encapsulation efficiency. For TPC, the formulation containing 0.5% extract (0.5 ENS) exhibited the highest encapsulation efficiency (70.88 ± 0.77%). For TFC, the formulations containing 0.4% (0.4 ENS) and 0.5% extract (0.5 ENS) achieved similarly high encapsulation efficiencies of 90.61 ± 0.17% and 90.49 ± 0.04%, respectively, with no significant difference. Furthermore, all extract-loaded formulations exhibited particle sizes below 200 nm and PDI values below 1.0, indicating a relatively uniform size distribution. In contrast, blank niosomes (BNS) displayed a significantly larger particle size of 333.63 ± 18.01 nm. In addition, all formulations showed zeta potential values more negative than −30 mV (Table 3). Overall, these findings indicate that a 0.5% *O. indicum* leaf extract is the optimal concentration for the niosomal formulation.

Encapsulation efficiency (%EE) was determined using total phenolic content (TPC) and total flavonoid content (TFC) to evaluate the overall retention of bioactive constituents in the crude *O. indicum* leaf extract, a commonly adopted approach for complex botanical extracts in which individual marker compounds are not isolated [19].

#### 3.1.3. Stability Study of Niosomes Under Temperature Cycling Conditions

The stability of blank niosomes (BNS) and five niosome formulations containing *O. indicum* leaf extract at concentrations ranging from 0.1 to 0.5% *w*/*v* (ENS) was evaluated under normal and heating-cooling conditions (Table 4). The results revealed formulation-dependent changes in encapsulation efficiency, particle size, PDI, and zeta potential.

For TPC, the percentage encapsulation efficiency (%EE) increased with extract concentration from 0.1% to 0.5%. The %EE of TPC ranged from 52.40% (0.1 ENS) to 70.75% (0.5 ENS). All formulations exhibited a decrease in %EE of TPC after storage. The %EE decreased significantly from 52.40% to 46.16% in 0.1 ENS and from 66.91% to 59.03% in 0.2 ENS. The %EE showed no significant difference in 0.3 ENS (63.60% to 63.02%), 0.4 ENS (68.55% to 67.43%), and 0.5 ENS (70.75% to 69.52%). This suggests that higher extract loading was associated with greater stability of phenolic encapsulation during storage.

For TFC, the %EE values were consistently higher than those of TPC across all formulations, indicating a greater affinity or compatibility of flavonoids with the niosomal system. The %EE of TFC ranged from 74.98% (0.1 ENS) to 90.70% (0.4 ENS). In contrast to TPC, the effect of storage on TFC was not consistent. Some formulations showed a decrease (0.2 ENS: 90.55% to 88.04%; 0.5 ENS: 90.58% to 88.66%), and others exhibited an increase (0.1 ENS: 74.97% to 80.73%; 0.3 ENS: 80.81% to 89.09%). These changes may be related to redistribution or enhanced encapsulation of flavonoids during storage.

Although all formulations exhibited high encapsulation efficiency for TFC, those containing higher extract concentration (0.3–0.5%) showed better stability, with no significant differences observed in TPC, particle size, and zeta potential. While TPC consistently declined during storage, TFC showed both decreases and notable increases, depending on the formulation. Among all formulations, 0.5 ENS demonstrated the highest and most stable %EE for TPC, followed closely by 0.4 ENS, indicating that higher extract concentrations favor both encapsulation efficiency and stability in the niosomal system.

All ENS formulations showed a marked increase in particle size after the heating–cooling cycle. For example, 0.1 ENS initially exhibited the smallest particle size of 124.63 ± 4.65 nm, which increased to 164.70 ± 1.40 nm after storage under specified conditions. In contrast, 0.5 ENS showed the largest initial particle size (188.97± 5.01 nm), which further increased to 289.13 ± 5.01 nm after storage. Furthermore, the zeta potential values became less negative after storage in most formulations, indicating a reduction in electrostatic stability. For example, 0.1 ENS changed from −44.50 to −27.53 mV. This decrease in the surface charge likely contributed to particle aggregation owing to the reduced repulsive forces between the vesicles. However, some formulations (e.g., 0.3 ENS, 0.4 ENS, and 0.5 ENS) showed relatively minor changes, suggesting better stability [20].

In addition, the PDI values increased after storage for all formulations, indicating a broader size distribution and reduced particle homogeneity. The PDI of 0.2 ENS and 0.3 ENS did not change significantly during storage, whereas 0.1 ENS, 0.4 ENS, and 0.5 ENS showed significant changes in PDI before and after storage. This observation further supports particle aggregation and reduced colloidal stability following thermal cycling.

Among the ENS formulations tested (0.1–0.5 ENS), 0.5 ENS exhibited the highest encapsulation efficiency for TPC (70.75 ± 0.77% before storage, 69.52 ± 1.15% after storage) and a comparably high encapsulation efficiency for TFC (90.58 ± 0.04% before storage, 88.66 ± 0.13% after storage). This formulation also showed the lowest PDI before storage (0.22 ± 0.01) among all formulations tested, and the smallest change in zeta potential after storage (from −39.50 ± 6.35 to −36.10 ± 0.26 mV), compared with the other ENS formulations. Therefore, 0.5% *O. indicum* leaf extract noisome (0.5 ENS) was selected for further characterization.

#### 3.1.4. Morphology by Transmission Electron Microscopy (TEM)

**Figure 1:**
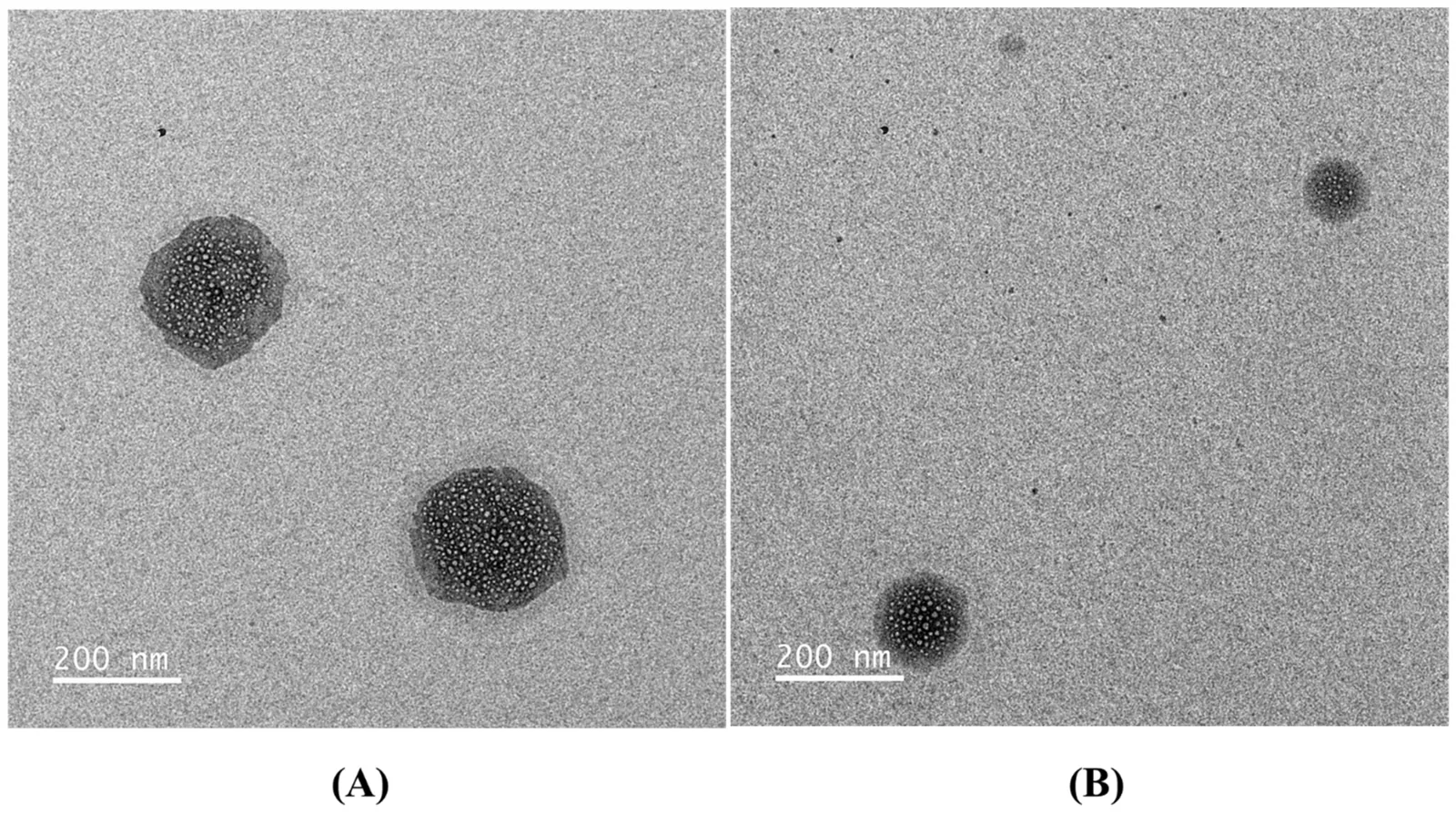
Morphology by transmission electron microscopy (TEM) images: blank noisome, BNS (**A**) and 0.5% *O. indicum* leaf extract noisome, ENS (**B**).

Both blank and loaded niosomes exhibited spherical morphologies (Figure 1). Notably, niosomes containing 0.5% *O. indicum* leaf extract (Figure 1B) displayed smaller particle sizes than blank niosomes (Figure 1A). According to previous studies, this is due to the bilayer arrangement of surfactant molecules, specifically the internal particle structure of Span 60, which is a surfactant with both hydrophilic and hydrophobic properties.

#### 3.1.5. ATR-FTIR Analysis

FTIR spectra of niosome compositions are shown in Figures S3 and S4. The FTIR spectrum of Span 60 (Figure S3) shows a broad peak at 3381.08 cm^−1^, indicating O-H stretching, peaks at 2915.11 and 2848.12 cm^−1^, indicating CH_3_-CH_2_ stretching, and peaks at 1732.66 and 1466.86 cm^−1^, 1172.40 and 720.63 cm^−1^, indicating C=O stretching, C-H bending, C-O-C stretching, and CH_2_ rocking, respectively. The FTIR spectrum of cholesterol (Figure S3) shows peaks at 3427.91 cm^−1^ due to OH stretching and peaks at 2928.36, 2898.65, 2864.56, and 2845.57 cm^−1^ indicating C-H stretching, peaks at 1462.81 and 1434.32 cm^−1^ indicating C-H bending, and peaks at 1053.85 and 1021.58 cm^−1^ indicating C-O-C and C-O stretching from alcohol, respectively [21].

Furthermore, the FTIR spectrum of *O. indicum* leaf extract (Figure S5), which contains some major compounds such as baicalin, baicalein, oroxylin A, and chrysin [5], shows a peak at 3306.23 cm^−1^ indicating OH stretching, peaks at 2922.15 and 2852.71 cm^−1^ indicating C-H stretching of methylene compounds, a peak at 1651.50 cm^−1^ typically associated with aromatic carbonyl groups (C=O stretching), which may suggest the presence of conjugated systems or specific aromatic functionalities in the extract, and peaks at 1605.36 and 1582.73 cm^−1^ characteristic of C=C stretching vibrations found in aromatic compounds, confirming their aromatic nature, and peaks at 1494.41, 1448.43, and 1413.09 cm^−1^ indicating C-H Bending in aromatic compounds. In addition, the peak at 1365.17 cm^−1^ indicates C-OH stretching, suggesting the presence of hydroxyl groups, which are common in phenolic compounds and alcohols. The peaks at 1283.56 and 1245.54 cm^−1^ indicate C-O stretching in ether or alcohol functionalities typical for flavonoids; the peak at 1157.58 cm^−1^ indicates C-O-C stretching, and the peaks at 1078.85 and 1029.62 cm^−1^ indicate C-O bending [22,23].

The FTIR spectrum of the blank niosome (BNS) in Figure S6 shows a strong broad peak at 3312.98 cm^−1^ for O-H stretching and peaks at 2966.46, 2926.02, and 2877.49 cm^−1^ for C-H stretching in the aliphatic chains (CH_3_-CH_2_) of Span 60 and cholesterol. The medium peak at approximately 1457.77 cm^−1^ can be attributed to C-C stretching. In addition, the strong peak at 1034.95 cm^−1^ in BNS is attributed to C-O stretching [24]. The peak at 1732.66 cm^−1^ for carbonyl groups, particularly from the ester functionalities in Span 60, was reduced to a very small peak, and a small new peak appeared at 1654.38 cm^−1^ in BNS (Figure S6). This indicates a reaction between the C=O of Span 60 and cholesterol molecules. Furthermore, peaks in the range of 1400–1500 cm^−1^ are commonly associated with C-C stretching, particularly in aliphatic and aromatic compounds. The presence of this peak suggests that the niosome formulation retained the structural characteristics of the surfactant and cholesterol. The medium peaks at 1457.77 and 1410.71 cm^−1^ can be attributed to C-C stretching vibrations.

**Figure 2:**
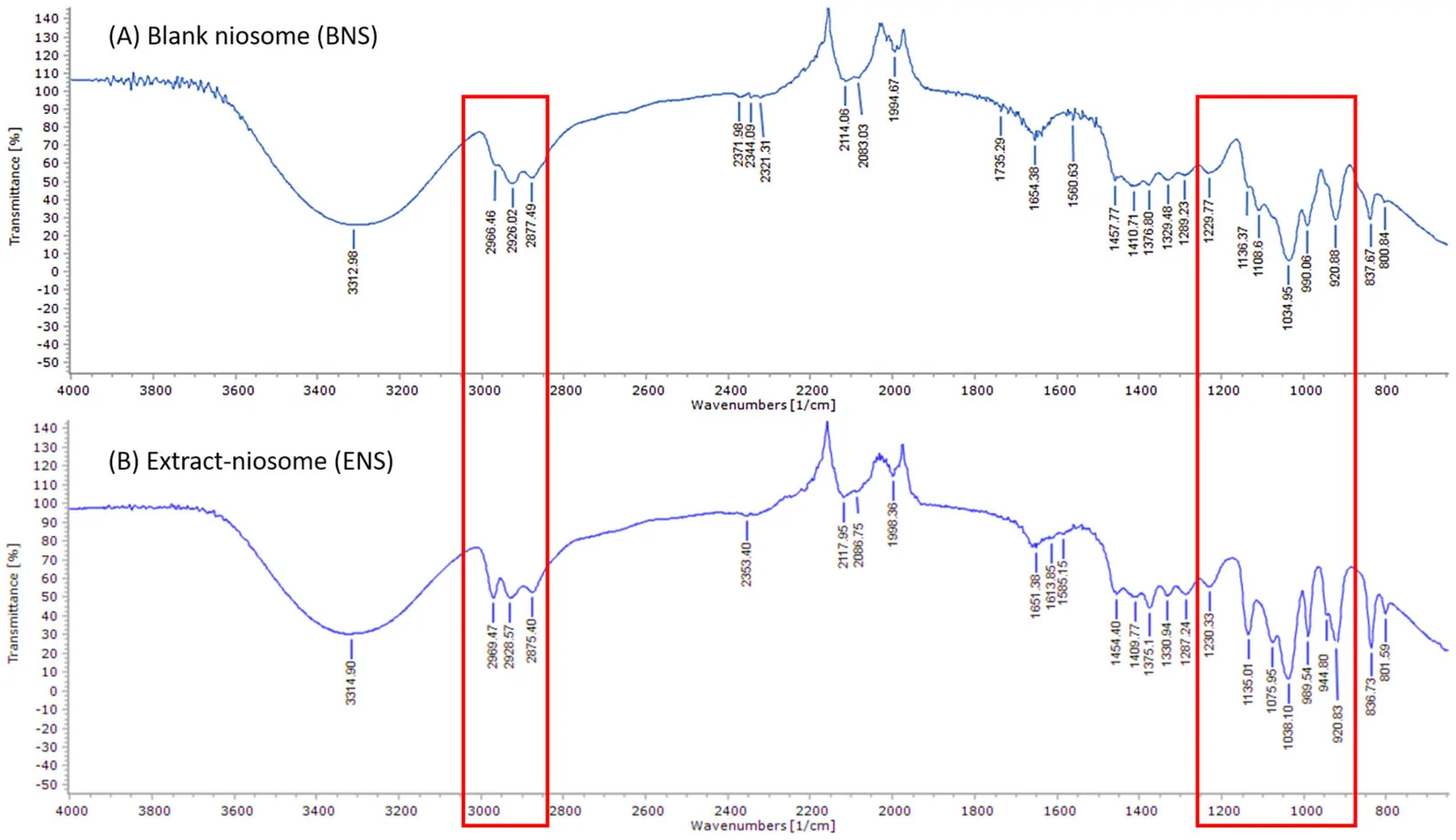
FTIR spectra of blank niosomes, BNS (**A**), and *O. indicum* leaf extract niosomes, ENS (**B**).

The FTIR spectrum of niosomes entrapped with *O. indicum* leaf extract (ENS), shown in Figure S7, presented some peaks similar to BNS, such as a strong broad peak at 3314.90 cm^−1^ for O-H stretching, the peaks at 2969.47, 2928.57, and 2875.40 cm^−1^ for C-H stretching in aliphatic chains (CH_3_-CH_2_) of Span 60 and cholesterol, the peak at 1651.38 cm^−1^ and the medium peaks at 1454.40 and 1409.77 cm^−1^. However, some peaks in the FTIR spectra of BNS and ENS showed different characteristics (Figure 2). The medium peak of the C-H stretching at 2969.47 cm^−1^ for ENS is stronger than that at 2966.46 cm^−1^ for BNS. In addition, the peak of C-O stretching at 1135.01 cm^−1^ for ENS is stronger than the peak at 1136.37 cm^−1^ for BNS, and the small peak at 1108.60 cm^−1^ observed in BNS is not observed in ENS. The strong peak at 1034.95 cm^−1^, corresponding to the C-O stretching of ether or ester functional groups in Span 60 (sorbitan monostearate) and cholesterol components within the niosomal formulation in BNS, was slightly shifted to 1038.10 cm^−1^ in the ENS formulation. These effects result from the interactions between the C-O-C groups in the extract encapsulated within the niosomes.

#### 3.1.6. Release Profile and Kinetic Model

**Figure 3:**
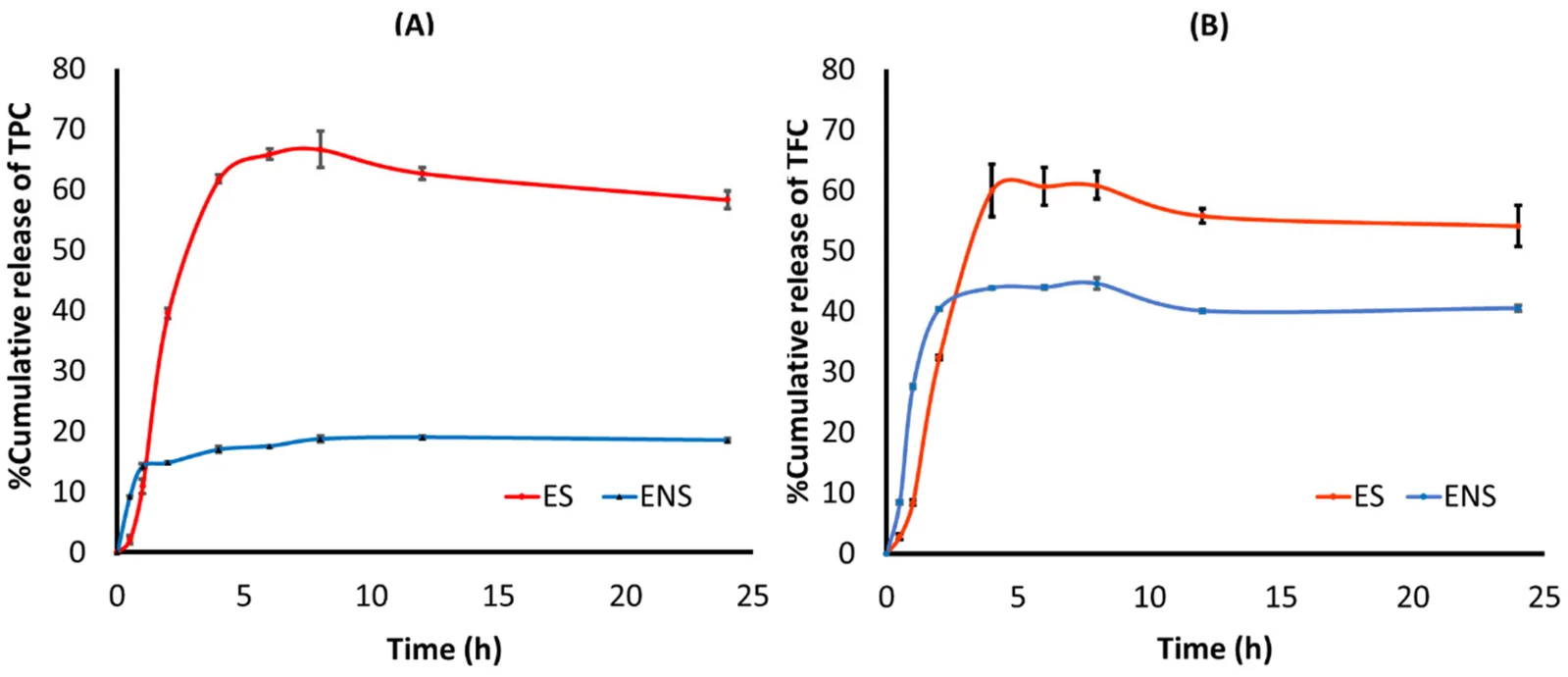
Cumulative release profile of TPC (**A**) and TFC (**B**) from *O. indicum* leaf extract niosome (ENS) compared with *O. indicum* extract solution (ES) obtained through a dialysis membrane using a Franz diffusion cell for 24 h. Data are presented as mean ± SD (*n* = 3).

The release profiles of total phenolic (TPC) and total flavonoid (TFC) from the extract solution (ES) and extract-loaded niosome (ENS) are presented in Figure 3A and Figure 3B, respectively. For TPC (Figure 3A), ES exhibited a rapid initial release, reaching approximately 60% within the first 4 h and peaking at 66% at 8 h. Thereafter, a slight decline was observed, and the release stabilized at 58% after 24 h. In contrast, ENS demonstrated a slower and more controlled release profile, with an initial release of 14% within the first 1 h and a sustained increase to 18% after 24 h. Similarly, for TFC (Figure 3B), ES demonstrated a pronounced burst release, up to 60% within 4 h, followed by a slight decline and stabilization at approximately 54% after 24 h. Conversely, ENS exhibited an initial release of approximately 40% in the first 2 h, after which the release profile remained stable at approximately 40% at 24 h. The markedly different release profiles of ES and ENS highlight the impact of vesicular encapsulation on modulating phytochemical release. In addition, the in vitro release profiles of the four markers (baicalin, baicalein, chrysin, and oroxylin A) were shown in Figure S8 to confirm that the extract-loaded niosome (ENS) exhibited a more sustained release pattern than the crude *Oroxylum indicum* extract solution (ES). These findings complement the overall release evaluation based on TPC and TFC by providing compound-specific information on the release behavior of representative flavonoid markers.

The release kinetics were evaluated using four models: zero-order (cumulative release percentage versus time), first-order (log cumulative drug release percentage versus time), Higuchi (cumulative drug release percentage versus square root of time), and Korsmeyer–Peppas (log cumulative drug release percentage versus log time). The correlation coefficients for all release models were determined by linear regression analysis. The Korsmeyer–Peppas model was used to analyze the release mechanism and diffusion kinetics [17]. The release exponent (*n*) was obtained from the slope of the linear regression in the log–log plot.

Kinetic modeling of TPC release (Table 5) demonstrated distinct release behaviors between ES and ENS. For ES, the release profile showed the highest correlation with the zero-order model (R^2^ = 0.954), followed by the Korsmeyer–Peppas model (R^2^ = 0.934). This suggests that TPC was released from the ES at a relatively constant rate, independent of its concentration. Although the Korsmeyer–Peppas model yielded a relatively high correlation, the corresponding release exponent (*n* = 1.559) should be interpreted with caution because the ES does not represent a matrix-controlled delivery system. Therefore, the mechanistic implications of this model for ES remain unclear.

For ENS, the Korsmeyer–Peppas model (R^2^ = 0.951) and the zero-order model (R^2^ = 0.951) gave comparable fits, both higher than Higuchi model (R^2^ = 0.852). The release exponent (*n* = 0.220), well below the threshold of 0.43 for spherical matrices, indicates a Fickian diffusion mechanism. This suggests that TPC release from niosomes is primarily controlled by diffusion through the vesicular bilayer rather than by relaxation of matrix structure.

A similar trend was observed for TFC (Table 6), with notable differences in the kinetic models describing ES and ENS. For ES, the release profile showed the best fit to the zero-order model (R^2^ = 0.984), with comparably high values for the Higuchi (R^2^ = 0.983) and Korsmeyer–Peppas model (R^2^ = 0.980). The release exponent (*n* = 0.933) exceeded 0.85. This indicates that TFC release from ES proceeded at a nearly constant rate, independent of drug concentration. The release exponent (*n* = 0.933) exceeded 0.85, a threshold conventionally used to indicate non-Fickian transport in matrix-controlled systems. However, similar to TPC, this parameter should be interpreted with caution because ES is a solution without vesicular structure and the value reflects the shape of the release curve, not an actual diffusion-relaxation mechanism.

For ENS, the release data were best fitted to the Higuchi model (R^2^ = 0.819), with a comparable fit to the Korsmeyer–Peppas model (R^2^ = 0.818). This suggests that diffusion contributed substantially to TFC release from the niosomal matrix. The release exponent (*n* = 1.287) exceeded 1, indicating a super Case II transport mechanism, in which polymer relaxation or reorganization of the niosomal bilayer controlled the release rate rather than diffusion alone. This behavior may be attributed to stronger interactions between flavonoid compounds and the vesicular membrane, leading to a more complex release mechanism than that observed for TPC.

#### 3.1.7. In Vitro Permeation of Phenolic Compounds

**Figure 4:**
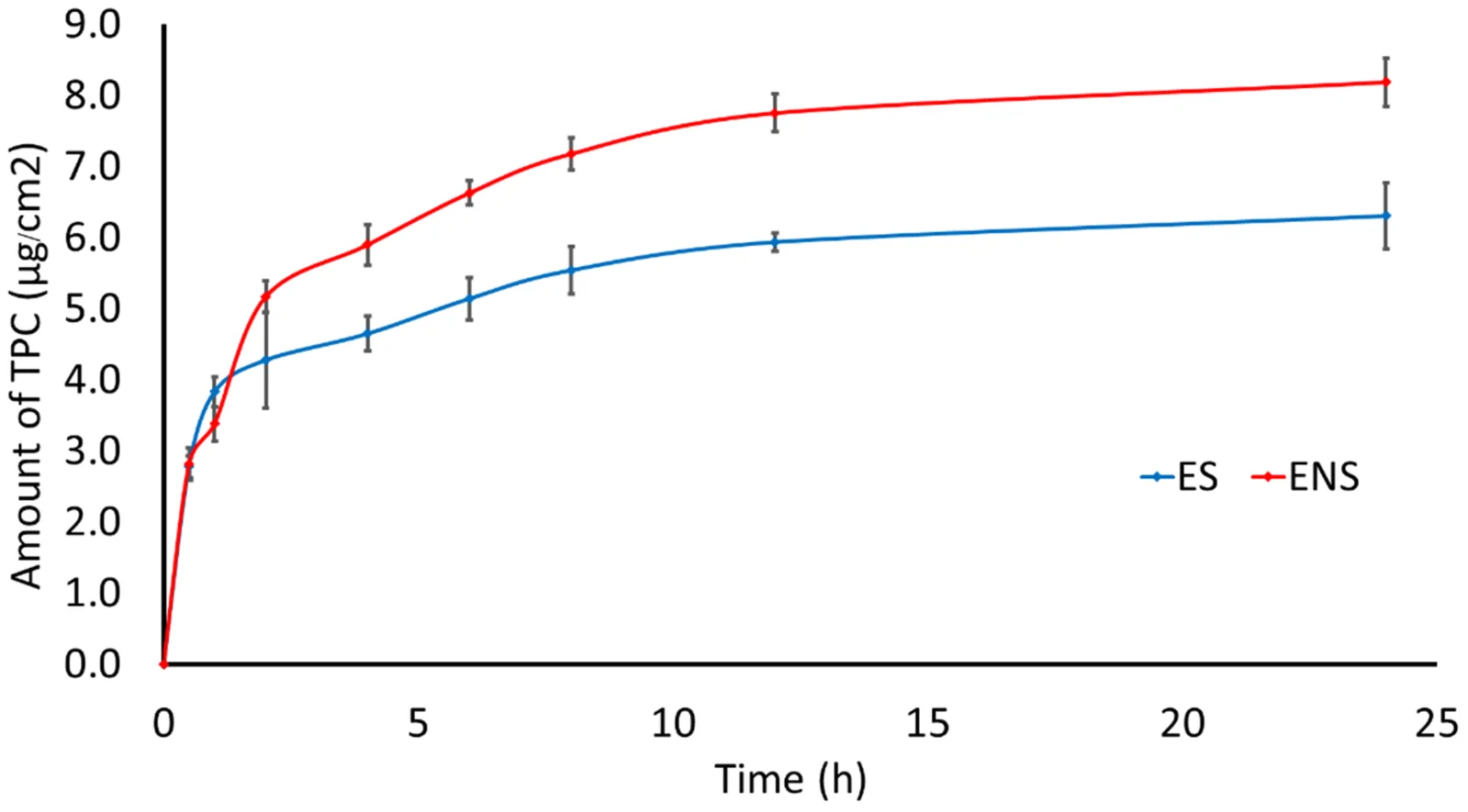
Permeation profile of total phenolic content (TPC) from *O. indicum* leaf extract niosomes (ENS) compared with *O. indicum* extract solutions (ES). The permeation profile of TPC was obtained using a Strat-M ^®^ membrane in a Franz diffusion cell for 24 h. Data are presented as mean ± SD (*n* = 3).

In the permeation study, total phenolic permeation from the entrapped niosomes (ENS) was analyzed using a Stat-M membrane and compared with that of the extract solution (ES). The permeation profiles and parameters clearly demonstrate the differences between ES and ENS. ENS exhibited a consistently higher cumulative permeation of TPC than ES over 24 h (Figure 4 and Table 7). The cumulative amount at 24 h (Q24) for ENS (8.185 ± 0.341 µg/cm^2^) was higher than that of ES (6.302 ± 0.462 µg/cm^2^), indicating enhanced permeation through the membrane. The steady-state flux (Jss) of ENS (0.338 ± 0.074 µg/cm^2^·h) was higher than that of ES (0.214 ± 0.086 µg/cm^2^·h), corresponding to an enhancement ratio (ER) of 1.58. This indicates that the niosomal formulation improved the permeation rate by approximately 58% compared to the extract solution. Similarly, the permeability coefficient (P) of ENS was higher than that of ES, further supporting improved membrane transport. Furthermore, ENS showed a shorter lag time (13.997 ± 4.363 h) than ES (20.540 ± 10.695 h), suggesting that the niosomal system facilitated faster membrane penetration. This reduction in lag time reflects the improved interaction between the formulation and membrane.

In the in vitro permeation study, TFC in both the niosomal formulation and extract solution remained below the detection limit under the experimental conditions. Therefore, only the TPC results are presented in Figure 4.

### 3.2. Anti-Inflammatory Activity in RAW 264.7 Cells

#### 3.2.1. Cytotoxicity of the Extract and the Formulations in RAW 264.7 Cells

**Figure 5:**
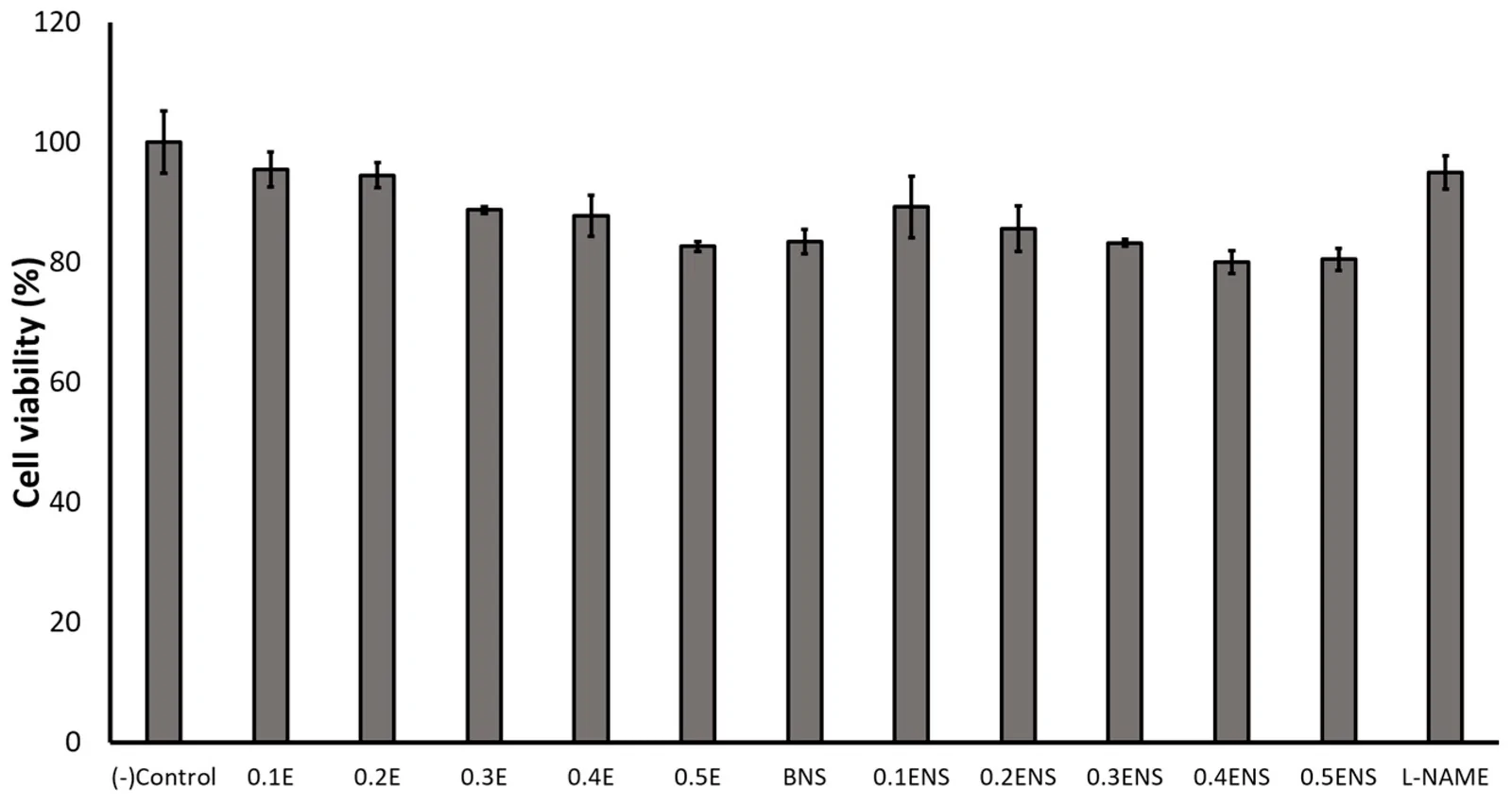
Cell viability of RAW 264.7 cells incubated for 24 h of untreated cells ((-) control) and RAW 264.7 cells treated with 0.1–0.5% extract solution (0.1–0.5 E), blank niosome (BNS), 0.1–0.5% extract niosome (0.1–0.5 ENS), and positive control 200 µM L-NAME (L-NAME). All Data are reported as mean ± SD (*n* = 3).

Cytotoxicity testing of *O. indicum* leaf extracts and entrapped niosomes was performed on RAW 264.7 cells using the MTT assay. Cells were exposed to the extract solutions and niosomes at varying concentrations of 0.1–0.5% for 24 h. The results demonstrated that the *O. indicum* leaf extracts at all concentrations, as well as their encapsulated niosomes, were non-cytotoxic, maintaining cell viability above 80% at all tested concentrations (Figure 5). However, increasing the concentration of the free extract increased cytotoxicity in a dose-dependent manner.

#### 3.2.2. Nitric Oxide Inhibition Activities

**Figure 6:**
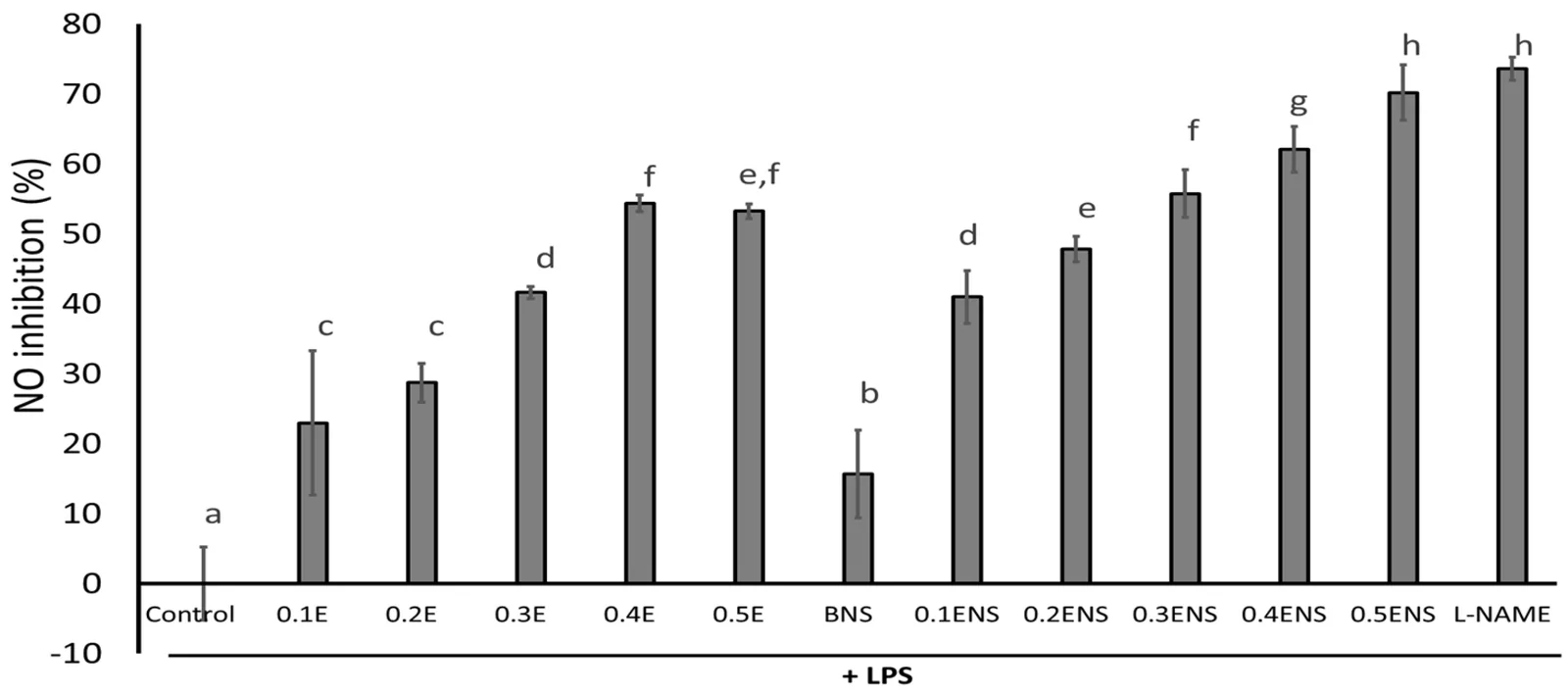
NO inhibition of LPS-stimulated RAW 264.7 cells (control), LPS-stimulated RAW 264.7 cells treated with 0.1–0.5% extract solution (0.1–0.5 E), blank niosome (BNS), 0.1–0.5% extract niosomes (0.1–0.5 ENS), and positive control 200 µM L-NAME (L-NAME). Different letters (a–h) indicate statistically significant differences between the groups (*p* < 0.05). All Data are reported as mean ± SD (*n* = 3).

The anti-inflammatory potential of *O. indicum* extracts was assessed by measuring their inhibitory effects on nitric oxide (NO) production in lipopolysaccharide (LPS)-stimulated RAW 264.7 macrophages. Following a 24 h incubation with 100 ng/mL LPS, niosomal formulations at all tested concentrations and *O. indicum* extracts at 0.1–0.5% significantly reduced NO production compared with LPS-treated cells, as shown in Figure 6.

In addition, a dose-dependent effect on NO inhibition was observed, with higher extract concentrations resulting in greater inhibition. Notably, 0.5% *O. indicum* extract exhibited the highest NO inhibition without cytotoxicity. All extract-loaded niosomal formulations exhibited greater NO inhibition than blank niosomes (BNS) in LPS-stimulated RAW 264.7 cells.

## 4. Discussion

The present study was designed to evaluate the overall performance of the multicomponent *O. indicum* extract following niosomal encapsulation rather than the behavior of individual phytochemical constituents. Botanical extracts are inherently complex mixtures containing multiple bioactive constituents, and their quality and biological properties are commonly evaluated using both global phytochemical parameters and selected marker compounds [25]. Therefore, total phenolic content (TPC) and total flavonoid content (TFC) were employed as global indicators to assess the collective retention, release, permeation, and stability of the botanical extract.

To obtain niosomes with optimal characteristics, key formulation variables including pH, the type of non-ionic surfactants, and the choice of co-solvents were evaluated. The performance of niosomes depends on the ionization behavior of the major bioactive constituents in the extracts, which tend to dissociate more effectively under alkaline conditions. At lower pH levels, reduced dissociation favors the partitioning of these compounds into the hydrophobic region of niosomal bilayers [26]. Consequently, decreasing alkalinity enhanced the encapsulation efficiency. In addition, the zeta potential depends on the ionization state of the compounds at different pH values and the type of additives used [27]. Previous findings showed that *Garcinia mangostana* extract-loaded niosomes exhibited a more negative zeta potential, attributed to the adsorption of ionized acidic groups from the extract onto the niosome surface [8].

In this study, Span 60 niosomes exhibited higher encapsulation efficiency than those prepared with Span 20 or Span 80 because their saturated C18 chain and higher phase transition temperature (Tc = 53 °C) generate a more rigid, less permeable bilayer that minimizes leakage of the moderately water-soluble extract [26]. Moreover, the presence of double bonds in Span 80 induces structural distortions in the hydrocarbon chains, leading to a more disordered and leaky bilayer structure [28].

The surfactant molecules align themselves in water such that the hydrophilic heads face the water and the hydrophobic tails align with each other, forming a bilayer and resulting in a spherical structure [29]. This variation in size is due to reduced repulsive forces between surfactant molecules, allowing them to pack more closely together. Consequently, the niosome extract was smaller than the blank niosome [30]. Furthermore, fluconazole-loaded niosomes formulated with Span 60 have been reported to exhibit higher encapsulation efficiency than those prepared with Span 20 or Span 80 [31]. Therefore, considering the HLB value, hydrocarbon chain length, and saturation, Span 60 was the most suitable surfactant for the formulation of the extract.

The effects of co-solvents indicated that formulations containing PEG400 had a lower ability to encapsulate phenolic and flavonoid compounds within niosomes. In contrast, formulations with PG exhibited significantly higher encapsulation efficiencies than those with PEG400. PG and PEG400 were incorporated as co-solvents and skin penetration enhancers in the formulations. They improved the solubility and dispersion of the extract within the hydrated niosomal system and may potentially contribute to improved dermal delivery of the entrapped bioactive compounds [32].

The reduction in particle size was also attributable to the use of polyols as bilayer modifiers. PG and PEG400 are known to interact with the surfactant bilayer by inserting into the polar head group region and partially penetrating the hydrophobic interface, thereby increasing bilayer fluidity and elasticity and enhancing the flexibility of the bilayer structure. Previous studies demonstrated that propylene glycol liposomes, formulated as PG-phospholipid vesicles or flexible liposomes, enhanced transdermal drug delivery by increasing membrane fluidity. This increased flexibility facilitates tighter vesicle packing, thereby reducing the particle size [30]. These results demonstrate that the use of 10% PG in the formulation provided optimal encapsulation efficiency and particle size of niosomes. Furthermore, PG interacts with surfactant molecules within the niosomal system, potentially altering their surface charge and consequently modulating the zeta potential of the nanoparticles [33].

The heating–cooling cycles increased the vesicle size and PDI and reduced the zeta potential, indicating partial destabilization of the niosomes. The increase in vesicle size suggests possible aggregation or fusion induced by repeated temperature fluctuations, which affect bilayer stability. However, the changes in most of the physical properties were relatively small, indicating that the overall structural integrity was largely preserved. These findings indicate that niosomes exhibit moderate stability under thermal stress, with some formulations demonstrating greater resistance to physicochemical changes. Following the heating–cooling cycle test, a reduction in encapsulation efficiency was observed, suggesting partial leakage of the encapsulated phytochemicals during storage. This reduction may be associated with partial destabilization of the niosomal bilayer and vesicle aggregation induced by thermal stress, as previously reported for niosomal systems [34]. Furthermore, the non-ionic surfactants used in niosomes make them less reactive with other components, leading to higher stability of the encapsulated active ingredients and longer half-life [35].

However, this present study evaluated only using the heating–cooling cycle test, which provides preliminary information on formulation stability under thermal stress. However, this approach does not replace long-term real-time or accelerated stability studies required for pharmaceutical product development and shelf-life determination according to the ICH Q1A(R2) guideline [36].

The optimal formulation 0.5% *O. indicum* leaf extract noisome (0.5 ENS) was selected for further characterization based on its highest encapsulation efficiency for both TPC and TFC among the ENS series, together with the lowest initial PDI and the most stable zeta potential upon storage.

The release profile of ES exhibited a rapid concentration-independent release for both TPC and TFC, as indicated by zero-order kinetics, reflecting the absence of diffusional barriers in the solution system. In contrast, for ENS (0.5 ENS), TPC release followed Fickian diffusion (*n* = 0.220), whereas TFC release followed a Super Case II mechanism (*n* = 1.287). These data indicate that both phenolics and flavonoids are predominantly released by a diffusion-controlled mechanism, with different best-fit models reflecting small differences in how each compound interacts and diffuses through the niosomal matrix. Niosomal encapsulation modulated the release of bioactive compounds according to their affinity for the vesicular membrane. The vesicle retained lipophilic flavonoids within the bilayer while allowing more hydrophilic phenolics to diffuse freely through the vesicle structure [37].

The niosome formulation demonstrated sustained release of phenolics and flavonoids for 24 h. This behavior can be attributed to the enhanced encapsulation of phenolic and flavonoid compounds within the surfactant-based bilayer of the niosome vesicles, which contributes to their controlled release [38]. The slight decline in cumulative TPC release observed for ES after 8 h (Figure 3A) may be attributed to physicochemical factors, including hydrolytic degradation and oxidation of phenolic compounds in the release medium, as well as analytical variability associated with TPC determination during extended incubation [39]. This behavior suggests that ES may exhibit limited physicochemical stability, whereas encapsulation in ENS provides partial protection.

Although the Korsmeyer–Peppas model was applied to characterize the release behavior. The formulation contained a complex herbal extract composed of multiple phytochemicals rather than a single active compound. Therefore, the calculated release exponent (*n*) should be interpreted as describing the overall release characteristics of the extract from the niosomal system rather than the release mechanism of individual constituents. The observed *n* values suggest that the release behavior may involve multiple contributing processes; however, these values should be interpreted cautiously because they represent the collective release profile of the complex extract rather than a single bioactive compound [40].

For in vitro permeation study, 40% *v*/*v* ethanol in phosphate buffer (pH 7.4) was used as the receptor phase to maintain sink conditions and obtain reliable permeation data for the poorly water-soluble extract rather than to replicate the exact skin environment. Under these hydroalcoholic conditions, the permeability coefficients are apparent, method-dependent values that are useful for comparing formulations within this in vitro model but should not be suitable for direct extrapolation to in vivo human skin.

According to a previous study, bioactive compounds encapsulated in niosomes exhibited higher permeation than those in the solution without niosomes. This enhancement is attributed to interactions between the active ingredients and the membrane surface, leading to a greater membrane permeation mechanism [41]. The permeation study used the Strat-M synthetic membrane rather than excised animal or human skin. Although Strat-M is widely used and correlates well with biological skin, it cannot fully reproduce the structural and physiological complexity of human skin. Therefore, confirmation using excised animal or human skin would be required to validate the permeation performance of the optimized formulation [42].

The niosomal formulation (ENS) exhibited significantly enhanced permeation compared to the extract solution (ES), as evidenced by a higher flux, permeability coefficient, and cumulative permeation (Q24), and shorter lag time. This improvement can be attributed to the vesicular structure of niosomes, which promotes interactions with the membrane and enhances drug partitioning. Additionally, the presence of non-ionic surfactants may increase membrane fluidity, facilitating the diffusion of phenolic compounds. The sustained-release behavior of ENS further supports the maintenance of the concentration gradient, thereby improving permeation. These findings confirm that niosomal encapsulation is an effective strategy for enhancing the transmembrane delivery of phenolic compounds [43].

For the cell assay using RAW 264.7 cells, a higher concentration of free extract increased cytotoxicity, likely due to the interaction of the extract with the cell membrane, leading to a higher cell death rate at higher concentrations. In contrast, the reduced cytotoxicity observed for the niosomal formulation may be associated with the cellular uptake of niosomes through endocytic pathways, potentially reducing the direct interaction of the extract with the cell membrane [44].

Nitric oxide (NO) is a key pro-inflammatory mediator produced by activated macrophages through inducible nitric oxide synthase (iNOS) and is one of the most widely used biomarkers for the preliminary evaluation of anti-inflammatory activity in LPS-stimulated RAW 264.7 macrophages [45]. Therefore, NO inhibition was selected as the initial biological endpoint in the present study to evaluate whether the developed niosomal formulation retained the NO inhibitory activity of the encapsulated *O. indicum* extract.

The results indicated that the niosome-encapsulated *O. indicum* extract was more effective in suppressing nitric oxide production than the non-encapsulated extract. The enhanced NO inhibitory activity may be attributed to more efficient intracellular delivery of the encapsulated extract, consistent with the endocytic uptake pathway discussed above [44].

Previous studies have demonstrated that *O. indicum* extract suppresses not only NO production but also several pro-inflammatory cytokines and inflammatory mediators, including TNF-α, IL-6, IL-1β, COX-2, and iNOS [46]. However, these biomarkers and signaling pathways were not entirely evaluated for the developed niosomal formulation in the present study. The anti-inflammatory findings reported here should be regarded as an initial evaluation based on NO inhibition. Further investigations involving inflammatory cytokines, inflammatory mediators, and signaling pathways, including NF-κB, are required to provide a more comprehensive understanding of the anti-inflammatory mechanisms of the developed niosomal formulation.

## 5. Conclusions

In the present study, niosomal formulations entrapping *O. indicum* leaf extract were successfully developed at pH 5.5 using Span 60 as the surfactant and 10% PG as the co-solvent. The incorporation of *O. indicum* extract during the rehydration step resulted in the highest retention, appropriate particle size, narrow size distribution, and suitable zeta potential. The optimized extract-loaded niosomes were spherical and approximately 100–200 nm in diameter, which was smaller than the corresponding blank niosomes (300–400 nm in diameter). The noisomal formulation exhibited sustained release of phenolic compounds over 6–24 h and flavonoids over 12–24 h. Phenolic permeation was also higher than the extract solution at the same concentration, indicating improved delivery across the membrane model.

Additionally, the stability study of the formulation, particularly at a high extract concentration (0.3–0.5%), showed no significant differences in the encapsulation efficiency of TPC, particle size, and zeta potential within acceptable limits. FTIR analysis confirmed the successful incorporation of *O. indicum* extract into the niosomal bilayer.

Furthermore, in vitro anti-inflammatory evaluation demonstrated that niosomes entrapping the *O. indicum* leaf extract exhibited lower cytotoxicity and greater inhibition of nitric oxide (NO) production than the free extract at equivalent concentrations, with the 0.5% niosomal formulation showing the strongest NO inhibition. This enhanced biological activity depends on the ability of niosomes to improve cellular uptake and local drug availability, while reducing off-target toxicity [35].

This niosome-based delivery system shows potential for commercial applications. However, further studies should investigate the effects of the niosome delivery system on inflammation inhibition through other mechanisms, such as the suppression of various cytokines and the regulation of COX-1 and COX-2 gene expression. Furthermore, investigations into the anti-inflammatory effects and potential side effects, including irritation, in animal models and clinical trials should be conducted before further development of commercial products.

This study has several limitations. Quantification of the four flavonoid markers (baicalin, baicalein, chrysin, and oroxylin A in niosome formulation was performed only for the initial content of the optimized niosomal formulation (Table S4). Encapsulation efficiency, release, permeation, and stability were instead monitored using total phenolic content (TPC) and total flavonoid content (TFC). The in vitro release, permeation, and stability determinations were performed using TPC and TFC, while the release study also investigated the four chemical markers. Permeation was evaluated using a Strat-M^®^ synthetic membrane rather than excised human or animal skin, and anti-inflammatory activity was assessed only through nitric oxide inhibition in LPS-stimulated RAW 264.7 cells. Therefore, the chemical markers permeation analysis, ex vivo or in vivo skin permeation studies, and evaluation of additional inflammatory biomarkers (e.g., TNF-α, IL-6, IL-1β, COX-2, iNOS) should be addressed in future work for topical application.

## 6. Patents

This work is protected under Petty Patent Application No. 2603000236, Department of Intellectual Property, Thailand, covering the niosomal formulation of *O. indicum* leaf extract described in this work.

## Figures and Tables

**Table 1:** The composition of niosome formulations.

Formulation	Condition	Component										
		CE (mg)	Chol (mg)	SP20 (mL)	SP60 (mg)	SP80 (mL)	PEG400 (mL)	PG (mL)	DI	pH (mL)		
										5.5	6.5	7.4
F1	DI	10	0.38	-	0.43	-	0.5	-	9.5	-	-	-
F2	pH 5.5	10	0.38	-	0.43	-	0.5	-	-	9.5	-	-
F3	pH 6.5	10	0.38	-	0.43	-	0.5	-	-	-	9.5	-
F4	pH 7.4	10	0.38	-	0.43	-	0.5	-	-	-	-	9.5
F5	SP20	10	0.38	0.35	-	-	0.5	-	-	9.5	-	-
F6	SP60	10	0.38	-	0.43	-	0.5	-	-	9.5	-	-
F7	SP80	10	0.38	-	-	0.43	0.5	-	-	9.5	-	-
F8	5% PEG400	10	0.38	-	0.43	-	0.5	-	-	9.5	-	-
F9	10% PEG400	10	0.38	-	0.43	-	1.0	-	-	9.0	-	-
F10	5% PG	10	0.38	-	0.43	-	-	0.5	-	9.5	-	-
F11	10% PG	10	0.38	-	0.43	-	-	1.0	-	9.0	-	-
F12 ^$^	10% PG	10	0.38	-	0.43	-	-	1.0	-	9.0	-	-

Crude extract; CE corresponds to a final concentration of 0.1% (*w*/*v*), Span 20; SP20, Span 60; SP60, Span 80; SP80, Cholesterol; Chol, Polyethylene glycol 400; PEG400, Propylene glycol; PG, PBS buffer pH 5.5; pH 5.5, PBS buffer pH 6.5; pH 6.5, PBS buffer pH 7.4; pH 7.4, Total phenolic content; TPC, Total flavonoid content; TFC. F12 ^$^, a rehydration solution containing the extract in 10 mL of 10% PG at pH 5.5, was used as a rehydration solution.

**Table 2:** Effect of pH, surfactant type, and co-solvent on the encapsulation efficiency (%EE) of total phenolic content (TPC) and total flavonoid content (TFC), particle size, polydispersity index (PDI), and zeta potential of *O. indicum* leaf extract-loaded niosomes.

Formulation	Condition	% Encapsulation Efficiency		Physical Properties		
		TPC	TFC	Size (nm)	PDI	Zeta Potential (mV)
F1	DI	20.44 ± 2.23 ^b^	35.65 ± 1.78 ^b^	262.63 ± 57.39 ^a^	1.00 ± 0.00 ^d^	0.97 ± 3.56 ^b^
F2	pH 5.5	31.38 ± 2.10 ^d^	57.34 ± 6.40 ^d^	201.33 ± 37.79 ^a^	0.49 ± 0.01 ^b^	−41.93 ±15.39 ^a^
F3	pH 6.5	25.54 ± 0.99 ^c^	45.38 ± 3.05 ^c^	282.20 ± 8.28 ^a^	0.69 ± 0.03 ^c^	−37.27 ± 1.40 ^a^
F4	pH 7.4	10.99 ± 0.71 ^a^	26.90 ± 1.27 ^a^	232.67 ± 53.46 ^a^	0.43 ± 0.04 ^a^	−44.60 ± 6.29 ^a^
F5	SP20	49.99 ± 1.45 ^b′^	61.05 ± 2.14 ^b′^	590.67 ± 18.93 ^c′^	1.00 ± 0.00 ^b′^	−58.47 ± 4.83 ^a′^
F6	SP60	46.46 ± 4.27 ^b′^	60.98 ± 1.56 ^b′^	292.33 ± 41.20 ^a′^	0.63 ± 0.10 ^a′^	−48.53 ± 3.88 ^b′^
F7	SP80	14.24 ± 10.85 ^a′^	18.13 ± 1.79 ^a′^	487.33 ± 45.17 ^b′^	1.00 ± 0.00 ^b′^	−34.57 ± 0.85 ^c′^
F8	5% PEG400	41.98 ± 2.06 ^b″^	67.21 ± 0.96 ^b″^	504.67 ± 33.50 ^d″^	0.45 ± 0.03 ^b″^	−50.10 ± 8.39 ^a″^
F9	10% PEG400	11.78 ± 2.94 ^a″^	24.31 ± 2.87 ^a″^	239.00 ± 2.48 ^b″^	0.24 ± 0.02 ^a″^	−27.87 ± 1.62 ^c″^
F10	5% PG	64.14 ± 1.40 ^c″^	81.51 ± 11.82 ^c″^	800.53 ± 14.48 ^e″^	0.68 ± 0.06 ^d″^	−36.20 ± 3.65 ^b″,c″^
F11	10% PG	70.55 ± 1.02 ^d″^	86.12 ± 8.51 ^c″^	277.80 ± 16.66 ^c″^	0.56 ± 0.07 ^c″^	−41.10 ± 10.10 ^a″,b″^
F12 ^$^	10% PG	67.98 ± 1.17 ^c″^	89.52 ± 3.53 ^c″^	174.50 ± 13.16 ^a″^	0.38 ± 0.09 ^b″^	−34.53 ± 1.15 ^b″,c″^

Crude extract; CE, Span 20; SP20, Span 60; SP60, Span 80; SP80, Cholesterol; Chol, Polyethylene glycol 400; PEG400, Propylene glycol; PG, PBS buffer pH 5.5; pH 5.5, PBS buffer pH 6.5; pH 6.5, PBS buffer pH 7.4; pH 7.4, Total phenolic content; TPC, Total flavonoid content; TFC. Data are expressed as mean ± SD (*n* = 3); values in the same column with different superscript letters a–d are significantly different (*p* < 0.05). Data are expressed as mean ± SD (*n* = 3). The values with superscript letters a–d are significantly different for F1–F4 (*p* < 0.05), a′–c′ are significantly different for F5–F7 (*p* < 0.05), and a″–e″ are significantly different for F8–F12 (*p* < 0.05) when compared between groups. F12 ^$^, a rehydration solution containing the extract in 10 mL of 10% PG at pH 5.5, was used as a rehydration solution.

**Table 3:** Effect of *O. indicum* leaf extract concentration on the encapsulation efficiency (%EE) of total phenolic content (TPC) and total flavonoid content (TFC), particle size, polydispersity index (PDI), and zeta potential of *O. indicum* leaf extract-loaded niosomes.

Formulation		Composition of Formulation					Encapsulation Efficiency (%)		Physical Properties		
		CE (mg)	Chol (mg)	SP60 (mg)	PG (mL)	pH 5.5 (mL)	TPC	TFC	Size (nm)	PDI	Zeta Potential (mV)
1	BNS	0.00	0.38	0.43	1.0	9.0	-	-	333.63 ± 18.01 ^c^	0.32 ± 0.03 ^c^	−38.17 ± 2.30 ^b^
2	0.1 ENS	10.00	0.38	0.43	1.0	9.0	52.83 ± 2.78 ^a^	74.78 ± 3.18 ^a^	124.63 ± 4.65 ^a^	0.24 ± 0.02 ^a,b^	−44.50 ± 4.23 ^a,b^
3	0.2 ENS	20.00	0.38	0.43	1.0	9.0	67.14 ± 1.31 ^c^	90.41 ± 0.76 ^c^	126.40 ± 7.45 ^a^	0.29 ± 0.02 ^c^	−44.33 ± 3.30 ^a,b^
4	0.3 ENS	30.00	0.38	0.43	1.0	9.0	63.78 ± 0.84 ^b^	80.72 ± 1.15 ^b^	176.30 ± 2.50 ^b^	0.29 ± 0.03 ^c^	−37.40 ± 6.74 ^b^
5	0.4 ENS	40.00	0.38	0.43	1.0	9.0	68.70 ± 1.05 ^c,d^	90.61 ± 0.17 ^c^	187.17 ± 3.35 ^b^	0.28 ± 0.02 ^b,c^	−52.77 ± 13.03 ^a^
6	0.5 ENS	50.00	0.38	0.43	1.0	9.0	70.88 ± 0.77 ^d^	90.49 ± 0.04 ^c^	188.97 ± 5.01 ^b^	0.22 ± 0.01 ^a^	−39.50 ± 6.35 ^a,b^

Crude extract; CE, Span 60; SP60, cholesterol; Chol, propylene glycol; PG, PBS pH 5.5; pH 5.5, total phenolic content; TPC, total flavonoid content; TFC. Data are expressed as mean ± SD (*n* = 3); values in the same column with different superscript letters ^a–d^ are significantly different (*p* < 0.05).

**Table 4:** Stability test of niosome formulations (ENS) based on particle size, PDI, Zeta potential, and %EE before and after storage conditions.

Formulation		Encapsulation Efficiency (%)				Physical Properties					
		TPC		TFC		Particle Size (nm)		PDI		Zeta Potential (mV)	
		Before	After	Before	After	Before	After	Before	After	Before	After
1	BNS	-	-	-	-	333.63 ± 18.01	364.77 ± 34.71	0.32 ± 0.03	0.42 ± 0.11	−38.17 ± 2.30	−7.21 ± 1.61 *
2	0.1 ENS	52.40 ± 2.81	46.16 ± 3.21 *	74.98 ± 3.15	80.73 ± 0.56	124.63 ± 4.65	164.70 ± 1.40 *	0.24 ± 0.02	0.33 ± 0.04 *	−44.50 ± 4.23	−27.53 ± 1.45 *
3	0.2 ENS	66.91 ± 1.32	59.03 ± 2.20 *	90.55 ± 0.75	88.04 ± 1.47 *	126.40 ± 7.45	211.03 ± 35.59	0.29 ± 0.02	0.35 ± 0.01	−44.33 ± 3.30	−35.00 ± 0.10 *
4	0.3 ENS	63.60 ± 0.84	63.02 ± 1.90	80.81 ± 1.15	89.09 ± 1.47 *	176.30 ± 2.50	235.33 ± 44.32	0.29 ± 0.03	0.33 ± 0.00	−37.40 ± 6.74	−35.87 ± 2.78
5	0.4 ENS	68.55 ± 1.05	67.43 ± 0.66	90.70 ± 0.17	88.69 ± 0.71 *	187.17 ± 3.35	250.33 ± 38.70	0.28 ± 0.02	0.51 ± 0.01 *	−52.77 ± 13.03	−35.00 ± 0.44
6	0.5 ENS	70.75 ± 0.77	69.52 ± 1.15	90.58 ± 0.04	88.66 ± 0.13 *	188.97 ± 5.01	289.13 ± 44.41	0.22 ± 0.01	0.72 ± 0.11 *	−39.50 ± 6.35	−36.10 ± 0.26

Crude extract: CE, Span 60; SP60, Cholesterol; Chol, Propylene glycol; PG, PBS buffer pH 5.5; pH 5.5, Total phenolic content; TPC, Total flavonoid content; TFC. Data expressed as mean ± SD (*n* = 3), * indicates a statistically significant difference compared with the before-storage condition (*p* < 0.05).

**Table 5:** Kinetic modeling of the in vitro release profile of total phenolic content (TPC) from *O. indicum* extract solution (ES) and *O. indicum* extract-loaded niosomes (ENS) using zero-order, first-order, Higuchi, and Korsmeyer–Peppas models.

Sample	Zero Order		First Order		Higuchi		Korsmeyer–Peppas		
	R^2^	*K*_0_	R^2^	*K*_1_	R^2^	*K_H_*	R^2^	*n*	*K_p_*
ES	0.954 ± 0.002	17.207 ± 0.306	0.752 ± 0.003	0.369 ± 0.001	0.913 ± 0.001	37.731 ± 0.702	0.934 ± 0.022	1.559 ± 0.150	0.937 ± 0.011
ENS	0.949 ± 0.024	0.648 ± 0.067	0.938 ± 0.022	0.017 ± 0.002	0.852 ± 0.017	29.755 ± 0.292	0.951 ± 0.031	0.220 ± 0.008	1.088 ± 0.005

R^2^: coefficient of determination; *K*_0_: zero-order release constant; *K*_1_: first-order release constant; *K_H_*: Higuchi release constant; *K_p_*: Korsmeyer–Peppas release constant; *n*: indicative of the release mechanism. All values are expressed as mean ± SD (*n* = 3).

**Table 6:** Kinetic modeling of the release profile of total flavonoid content (TFC) from *O. indicum* extract solution (ES) and *O. indicum* extract-loaded niosomes (ENS) using zero-order, first-order, Higuchi, and Korsmeyer–Peppas models.

Sample	Zero Order		First Order		Higuchi		Korsmeyer-Peppas		
	R^2^	*K*_0_	R^2^	*K*_1_	R^2^	*K_H_*	R^2^	*n*	*K_p_*
ES	0.984 ± 0.009	16.745 ±1.164	0.841 ± 0.026	0.355 ± 0.006	0.983 ± 0.002	46.191 ± 2.949	0.980 ± 0.008	0.933 ± 0.035	3.055 ± 0.089
ENS	0.705 ± 0.010	8.691 ± 0.087	0.568 ± 0.009	0.161 ± 0.002	0.819 ± 0.008	25.859 ± 0.214	0.818 ± 0.007	1.287 ± 0.006	1.538 ± 0.019

R^2^: coefficient of determination; *K*_0_: zero-order release constant; *K*_1_: first-order release constant; *K_H_*: Higuchi release constant; *K_p_*: Korsmeyer–Peppas release constant; *n*: indicative of the release mechanism.

**Table 7:** Comparison of the in vitro membrane permeation parameters of *O. indicum* leaf extract solution (ES) and *O. indicum* leaf extract-loaded niosomes (ENS) using a Franz diffusion cell system. Permeability parameters include flux, cumulative amount permeated at 24 h (Q24), enhancement ratio (ER), permeability coefficient (P), and lag time (T_lag_).

Formulations	Flux (µg/cm^2^·h)	Q24 (µg/cm^2^)	ER	P × 10^−3^ (cm^2^/h)	T_lag_ (h)
ES	0.214 ± 0.086	6.302 ± 0.462	1.00	0.107 ± 0.043	20.540 ± 10.695
ENS	0.338 ± 0.074	8.185 ± 0.341	1.58	0.187 ± 0.041	13.997 ± 4.363

Q24: cumulative amount permeated at 24 h; ER: enhancement ratio; P: permeability coefficient; T_lag_: Lag time; ES: *O. indicum* extract solution 0.5%; ENS: Extract niosome 0.5%. All values are presented as mean ± SD (*n* = 3).

## Data Availability

Data is contained within the article or Supplementary Materials.

## Supplementary Materials

The following supporting information can be downloaded at: https://www.mdpi.com/article/10.3390/pharmaceutics18080955/s1, Figure S1: HPLC chromatogram of the 4-standard mixture, Figure S2: Calibration curves of the 4 flavonoid markers in *Oroxylum indicum* extract, Figure S3: FTIR spectra of Span 60, Figure S4: FTIR spectra of cholesterol, Figure S5: FTIR spectra of *O. indicum* leaf extracts, Figure S6: FTIR spectra of Blank niosome (BNS), Figure S7: FTIR spectra of extract-niosome (ENS), Figure S8: In vitro cumulative release profiles of major flavonoid compounds from *O. indicum* extract, Table S1: Validation parameters of the HPLC method for the quantitative determination of baicalin, baicalein, chrysin, and oroxylin A, Table S2: Quantitative determination of the major flavonoid markers in *O. indicum* extract by HPLC, Table S3. Total phenolic content (TPC) and total flavonoid content (TFC) of the *O. indicum* var. nana extract used for niosome preparation, Table S4: Initial concentration of four major flavonoid markers in 0.5 ENS niosome formulation.

## Abbreviations

The following abbreviations are used in this manuscript:

ANOVA	Analysis of variance
ATR	Attenuated total reflectance
BNS	Blank niosomes
DLS	Dynamic light scattering
DMEM	Dulbecco’s modified Eagle’s medium
DMSO	Dimethyl sulfoxide
EE	Encapsulation efficiency
ENS	Extract-loaded niosomes
ER	Enhancement ratio
ES	Extract solution
FBS	Fetal bovine serum
FTIR	Fourier transform infrared spectroscopy
HLB	Hydrophilic-lipophilic balance
HPLC	High-performance liquid chromatography
JSS	Steady-state flux
L-NAME	N^ω^-nitro-L-arginine methyl ester
LPS	Lipopolysaccharide
MTT	3-(4,5-dimethylthiazol-2-yl)-2,5-diphenyltetrazolium bromide
NED	N-(1-naphthyl) ethylenediamine dihydrochloride
NO	Nitric oxide
P	Permeability coefficient
PBS	Phosphate-buffered saline
PDI	Polydispersity index
PEG400	Polyethylene glycol 400
PG	Propylene glycol
RAW 264.7	Murine macrophage cell line
SP20	Span 20
SP60	Span 60
SP80	Span 80
TEM	Transmission electron microscopy
TFC	Total flavonoid content
T/lag	Lag time
TPC	Total phenolic content
