# Supplementary material for: Niosomal Encapsulation of Oroxylum indicum Leaf Extract for Topical Anti-Inflammatory Application
Source: Pharmaceutics. 2026 Aug 3;18(8):955. doi: 10.3390/pharmaceutics18080955 (PMC13516058; doi:10.3390/pharmaceutics18080955)
Supplement: Supplementary file 1 [file pharmaceutics-18-00955-s001.zip › pharmaceutics-4471280-supplementary.pdf]

Supplementary Data

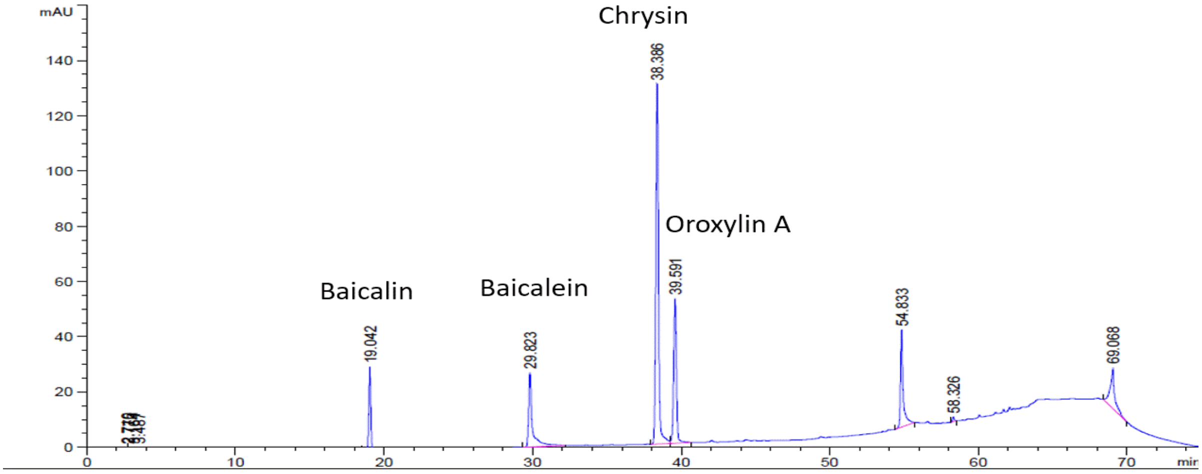

Figure S1. HPLC chromatogram of the 4-standard mixture of Baicalin (19.042 min), Baicalein (29.823 min), Chrysin (38.386 min) and Oroxylin A (39.591 min).

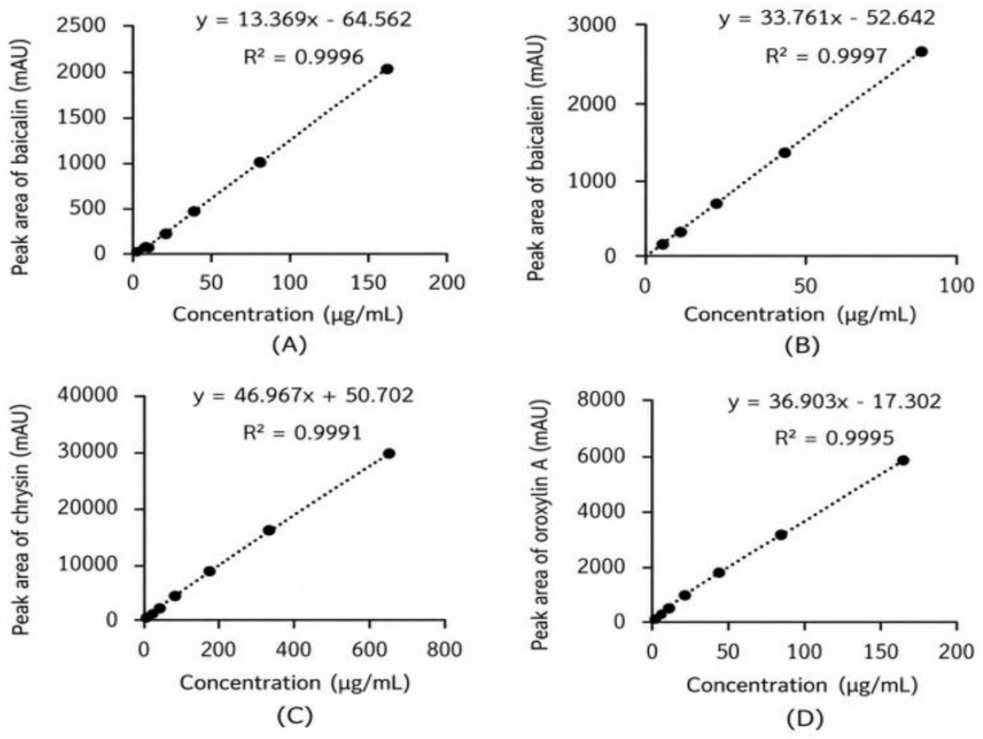

Figure S2. Calibration curves of the 4 flavonoid markers in *Oroxylum indicum* extract: (A) baicalin, (B) baicalein, (C) chrysin, and (D) oroxylin A used for quantitative analysis.

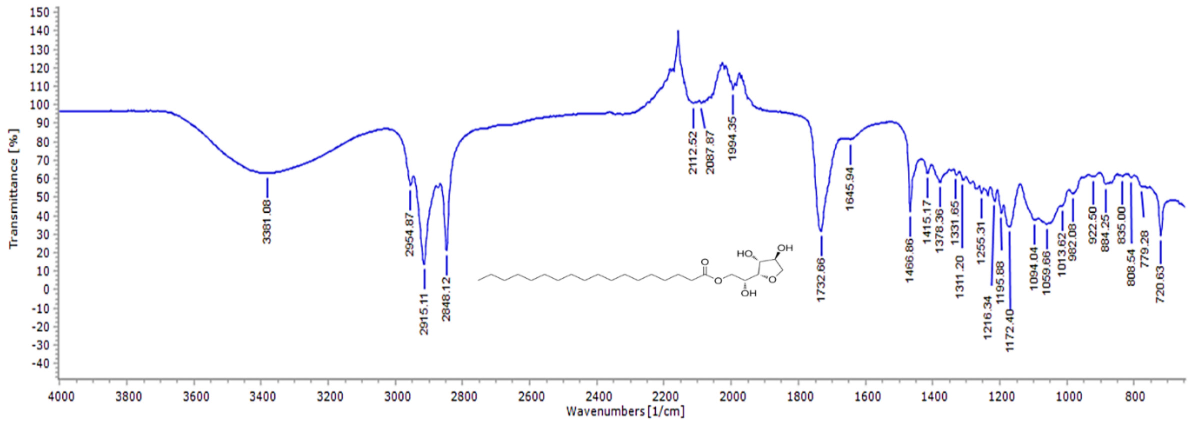

Figure S3: FTIR spectra of Span 60.

Span 60 (cm^{-1})	Functional groups
3381.08	Broad peak of O-H stretching
2915.11, 2848.12	CH_3-CH_2 stretching
1732.66	C=O stretching
1466.86	C-H bending
1172.40	C-O-C stretching
720.63	CH_2 rocking

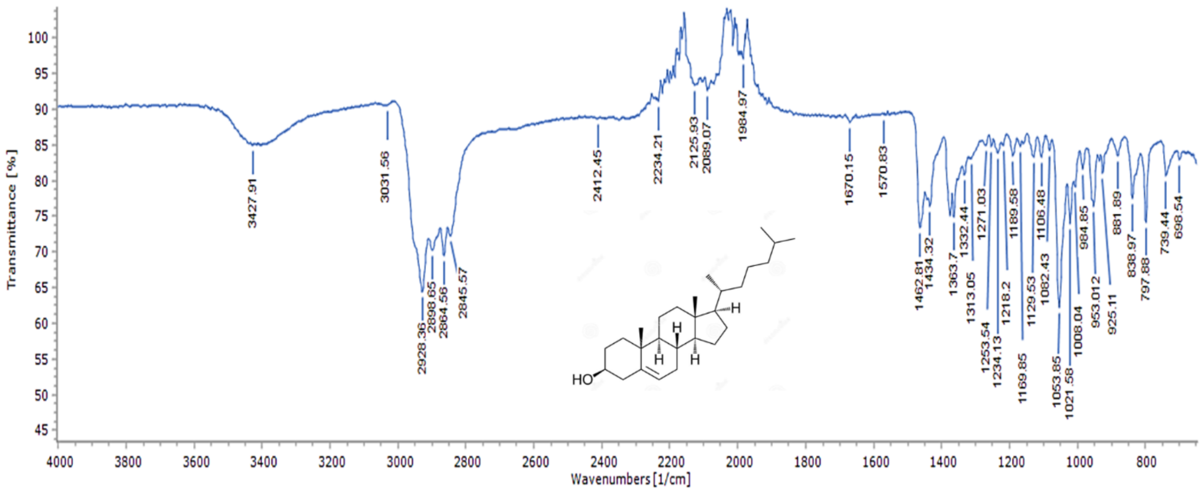

Figure S4: FTIR spectra of cholesterol.

Chloresterol (cm^{-1})	Functional groups
3427.91	Broad peak of O-H stretching
2928.36, 2898.65, 2864.56, 2845.57	CH_3-CH_2 stretching
1462.81, 1434.32	C-H bending
1053.85, 1021.58	C-O stretching of alcohol

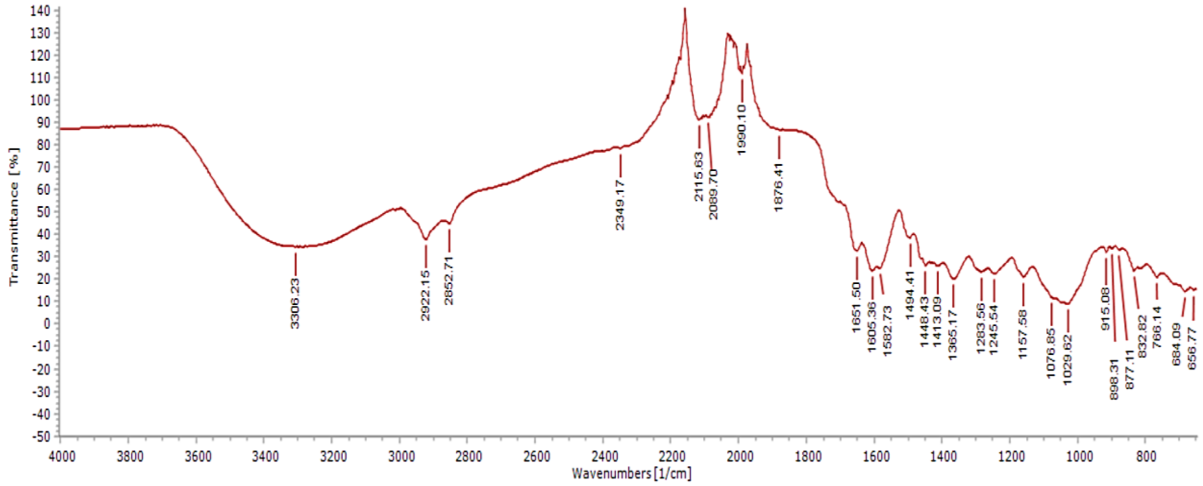

Figure S5: FTIR spectra of *O.indicum* leaf extracts (Zhou et al., 2017).

***O.indicum* leaf extracts (cm^{-1})**	**Functional groups**
(3200-3600) 3306.23	Broad peak of O-H stretching
2922.15, 2852.71	C-H stretching
1651.50	C=O Stretching
1605.36, 1582.73	C=C stretching (trans)
1494.41, 1448.43, 1413.09	C-H bending
1365.17	C-OH stretching
1283.56, 1245.54	C-O stretching
1157.58	C-O-C stretching
1078.85, 1029.62	C-O bending

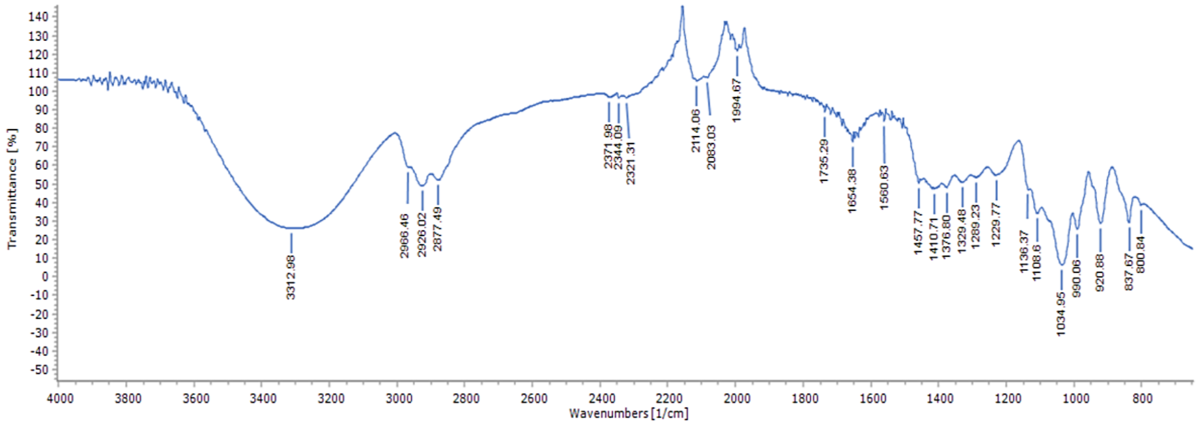

Figure S6: FTIR spectra of Blank niosome (BNS).

Blank niosome (cm^{-1})	Functional groups
3600-3000 (3312.98)	O-H stretching
2966.46, 2926.02, 2877.49	C-H stretching
**1654.38**	**C=C stretching**
1457.77, 1410.71	C-C stretching
1289.23, 1229.77	C-H bending
1034.95	C-O stretching?

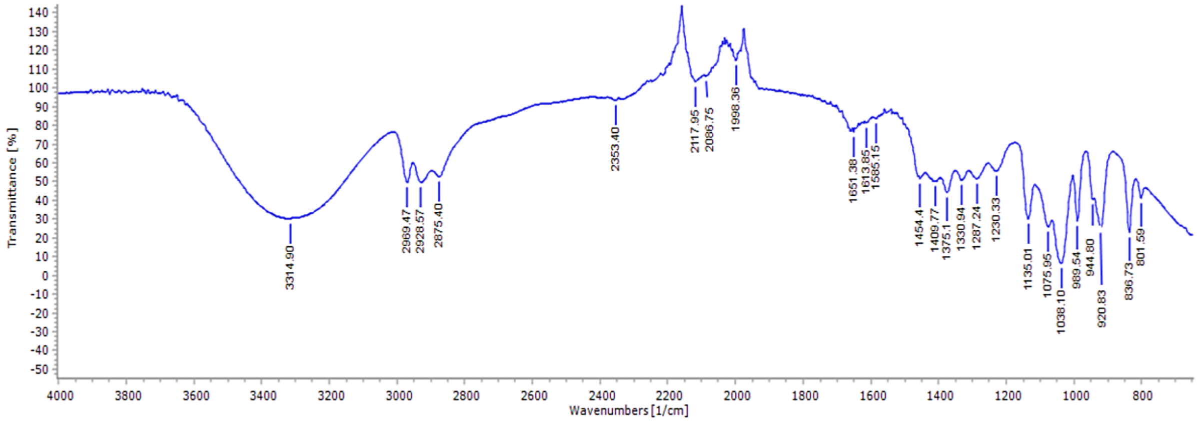

Figure S7: FTIR spectra of extract-niosome (ENS).

O.indicum **leaf extract niosome (cm^{-1})**	**Functional groups**
3314.90	O-H stretching
2969.47, 2928.57, 2875.40	CH_3-CH_2 stretching
1651.38	C=C stretching
1375.10, 1330.94	C-H bending ??
1135.01	C-O stretching??

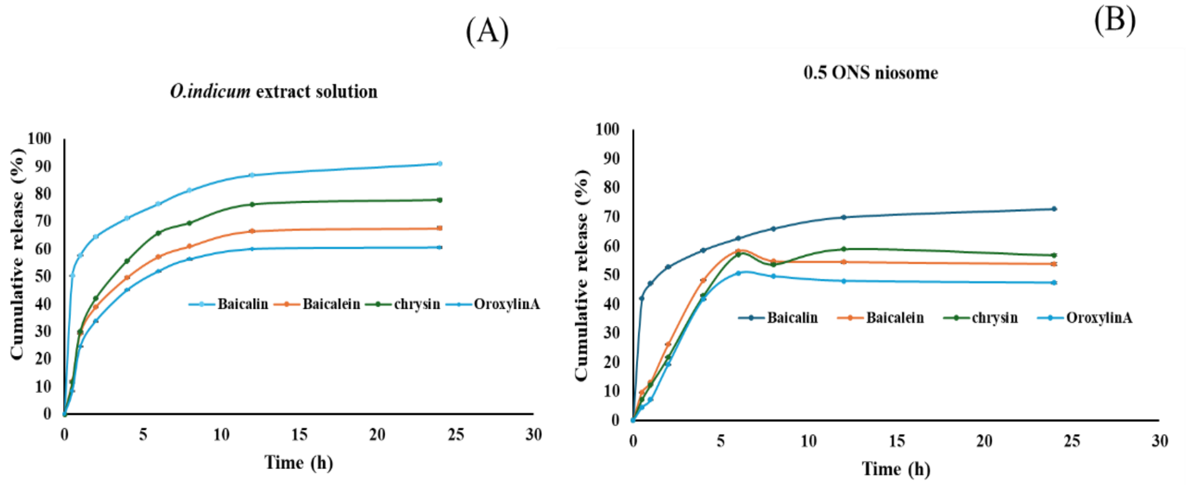

Figure S8. *In vitro* cumulative release profiles of major flavonoid compounds from *O. indicum extract*. (A) Cumulative release of baicalin, baicalein, chrysin, and oroxylin A from the *O. indicum* extract solution. (B) Cumulative release of the same compounds from 0.5% ONS-loaded niosomes over 24 h.

The extract solution exhibited a rapid initial release, whereas the niosomal formulation showed a more sustained release behavior, indicating the ability of niosomes to modulate and control the release of bioactive compounds.

Table S1: Validation parameters of the HPLC method for the quantitative determination of baicalin, baicalein, chrysin, and oroxylin A.

Validation parameter		Baicalin	Baicalein	Chrysin	Oroxylin A
**Linearity**	Concentration range (μg/mL)	5–160	5–80	5–640	5–160
	Regression equation	y = 13.369x - 64.562	y = 33.761x - 52.642	y = 46.967x + 50.702	y = 36.903x - 17.302
	Correlation coefficient (R^2)	R^2 = 0.9996	R^2 = 0.9997	R^2 = 0.9991	R^2 = 0.9995
**Sensitivity**	LOD (μg/mL)	2.5	1.0	0.5	1.0
	LOQ (μg/mL)	5.0	2.5	1.0	5.0
**Precision**	Intraday (%RSD)	0.99–1.86	0.05–1.53	0.60–1.14	0.79–1.41
	Interday (%RSD)	0.23–1.84	0.36–1.19	0.12–1.77	0.24–0.92
**Accuracy**	Accuracy (%Recovery)	97.01–104.39	96.76–106.76	95.45–100.76	99.98–109.24

Table S2: Quantitative determination of the major flavonoid markers in *Oroxylum indicum* extract by HPLC.

Compound	*Oroxylum indicum var. nana* (µg/mg extract)
**Baicalin**	33.44 ± 4.91
**Baicalein**	27.50 ± 8.23
**Chrysin**	5.20 ± 2.44
**Oroxylin A**	24.06 ± 7.20

Note. Values are presented as mean ± SD (n = 3).

Table S3. Total phenolic content (TPC) and total flavonoid content (TFC) of the *Oroxylum indicum var. nana* extract used for niosome preparation.

Analysis	*Oroxylum indicum var. nana* (mg/g DW)
Total phenolic content (TPC) (mg GAE/g DW)	15.96 ± 4.45
Total flavonoid content (TFC) (mg QE/g DW)	6.31 ± 1.96

Note. Values are presented as mean ± SE (N = 3). DW = dry weight.

Table S4: Initial concentration of four major flavonoid markers in 0.5 ENS niosome formulation

Compound	0.5 ENS (µg/mL)
**Baicalin**	59.33 ± 0.70
**Baicalein**	161.94 ± 1.86
**Chrysin**	75.69 ± 0.22
**Oroxylin A**	248.13 ± 0.71

Note. Values are presented as mean ± SD (n = 3).

Although the major flavonoids (baicalin, baicalein, chrysin, and oroxylin A) were characterized by HPLC before formulation to confirm the phytochemical profile of the extract, the TPC- and TFC-based approach does not distinguish the encapsulation efficiency of individual compounds, as each flavonoid may exhibit different encapsulation behaviors due to its physicochemical properties. Therefore, future studies employing HPLC quantification of individual marker compounds after

encapsulation would provide a more comprehensive evaluation of compound-specific encapsulation efficiency.

The 0.5% ENS niosomal formulation was also determined for 4 markers such as baicalin, baicalein, chrysin, and oroxylin A. The initial concentrations of baicalin, baicalein, chrysin, and oroxylin A in the 0.5% ENS niosomal formulation were quantified by a validated HPLC method (Table S4). Oroxylin A exhibited the highest initial concentration (248.13 ± 0.71 μg/mL), followed by baicalein (161.94 ± 1.86 μg/mL), chrysin (75.69 ± 0.22 μg/mL), and baicalin (59.33 ± 0.70 μg/mL). These initial concentrations were subsequently used as the baseline for the compound-specific cumulative release analysis presented in Figure S8.

To further characterize the release behavior of the major phytochemical constituents from 0.5% ENS niosomal formulation, the cumulative release profiles of baicalin, baicalein, chrysin, and oroxylin A were determined by HPLC (Figure S8). The crude *O.indicum* extract solution exhibited a relatively rapid initial release of all four marker compounds, followed by a gradual plateau. In contrast, the 0.5 ENS niosomal formulation showed a more sustained release pattern, indicating that niosomal encapsulation effectively modulated the release of the major flavonoid markers. Among the four compounds, baicalin exhibited the highest cumulative release, whereas oroxylin A showed the lowest release throughout the study period. These findings demonstrate that HPLC-based analysis of specific compounds complements the overall release data from TPC and TFC, giving additional insight into how representative flavonoid markers are released.
